# Full P_4_ to P^3−^ Reduction with a Redox‐Active Metal Crown Complex

**DOI:** 10.1002/anie.202515157

**Published:** 2025-09-01

**Authors:** Johannes Maurer, Marcel A. Schmidt, Michael Nägel, Christian Färber, Jens Langer, Sjoerd Harder

**Affiliations:** ^1^ Inorganic and Organometallic Chemistry Universität Erlangen Nürnberg Egerlandstrasse1 Erlangen 91058 Germany

**Keywords:** Anions, Crown, Low‐valent, Magnesium, P_4_ activation

## Abstract

Traditional bulk syntheses of phosphorus compounds start with P_4_ to PCl_3_ oxidation but more sustainable methods cleave P─P bonds reductively. This generally results in larger polyphosphide Zintl anions: P_m_^n^ˉ. We report a relatively selective full reduction of P_4_ at room temperature to give a unique hydrocarbon‐soluble *s*‐block metal complex of the P^3^ˉ anion. Key to this chemistry is a recently reported redox‐active metal crown complex: (BDI*)MgNa_3_N″_2_ (**VI**); N″ = N(SiMe_3_)_2_ and BDI* = HC[(*t*Bu)C═N‐DIPeP]_2_, DIPeP = 2,6‐CHEt_2_‐phenyl. The reduction of P_4_ according to 2 **VI** + 0.25 P_4_ → (BDI*)MgNa_5_N″_3_P (**1**) + 0.5 [(BDI*)Mg]_2_ + 0.33 (NaN″)_3_ is calculated to be exothermic (Δ*H* = −40.5 kcal mol^−1^). The crystal structure of **1** shows a strongly bound (BDI*)MgP^2^ˉ anion with two chelating [Na‐N″‐Na^+^] and [Na‐N″‐Na‐N″‐Na^+^] arms of unequal length. Although these arms are highly fluxional and rapidly exchange ions, they effectively stabilize the P^3^ˉ anion. DFT calculations confirm the highly ionic nature of the complex and describe P^3^ˉ as full valence‐shell anion with four lone‐pairs of electrons. Reactivity studies show that the P^3^ˉ anion can react as a triple Brønsted base, a three‐fold nucleophile or as a reducing agent.

## Graphical abstract

**Breaking the crown**: Reduction of P_4_ generally results in larger polyphosphide Zintl anions: P_m_^n^ˉ. However, using a hydrocarbon‐soluble Mg^0^ complex, P_4_ can be fully reduced to mononuclear P^3^ˉ. The metal crown opens to a two‐armed pincer‐like ligand that efficiently stabilizes the phosphido anion. Latter P^3^ˉ anion reacts as a base, a three‐fold nucleophile or a reducing agent.

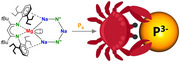

The traditional bulk syntheses of phosphorus compounds generally start with the oxidation of P_4_ to PCl_3_, using highly corrosive Cl_2_. More sustainable methods aim for metal‐mediated cleavage of P─P bonds,^[^ ^1^, ^2^, ^3^, ^4^ ^]^ photocatalysis,^[^ ^5^ ^]^ or oxidative functionalization.^[^ ^6^ ^]^ Reductive P_4_ conversion generally results in formation of polyphosphorus metal complexes with a large diversity of reduced P_n_ ligands.^[^ ^7^ ^]^ Terminal (M≡P) or bridging (M═P═M) phosphido ligands are rare and only found for the more covalently bound transition metals.^[^ ^8^ ^]^ In actinide chemistry such species have been well studied for their unusual double and triple bonds.^[^ ^9^, ^10^ ^]^ Formally fully reduced Pˉ^III^ is also found in *p*‐block metal complexes like P(SnPh_3_)_3_^[^ ^11^ ^]^ or the Sn^II^ phosphide complex **I** (Scheme 1).^[^ ^12^ ^]^ However, even in complexes with the most electropositive *p*‐block metal Al (**II**‐**III**; Scheme 1),^[^ ^13^, ^14^ ^]^ the electronegativity difference is small (Al: 1.61, P: 2.19)^[^ ^15^ ^]^ and metal─P bonds are of polarized covalent nature. The most polar phosphido complex is likely represented by a large Y^III^ cluster [K^+^·(toluene)][Y_5_I_5_(PAr)_4_P¯·(THF)_4_] (**IV**) which contains an interstitial Pˉ^III^.^[^ ^16^ ^]^ Like **I**, complex **IV** was obtained by serendipitous P─SiMe_3_ bond cleavage rather than by reduction of elemental phosphorus.

**Scheme 1:**
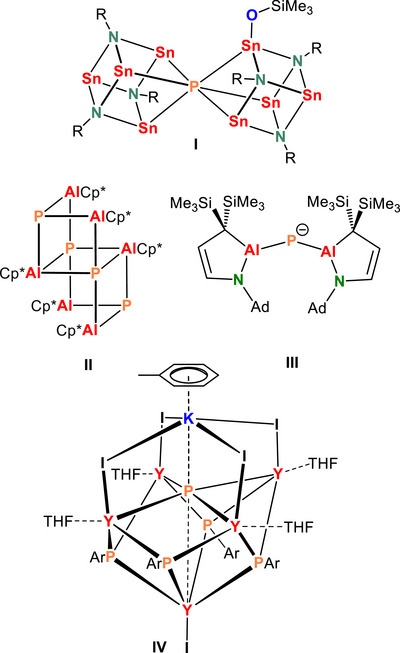
Examples of late main group metal complexes with the phosphido ligand (Pˉ^III^).

Hitherto, there are no reports on highly polar early main group metal complexes of the P^3^ˉ anion. Although this anion reaches a full valence shell and according to the 8‐electron rule should be stable, the considerable 3– charge on the phosphido ligand challenges the synthesis of highly ionic complexes. Inorganic *s*‐block metal Zintl phases like Na_3_P have been prepared from the elements at 700 °C.^[^ ^17^ ^]^ Using liquid ammonia as a highly polar solvent, white or even red phosphorus can be easily reduced by Na at much lower temperature but this results in a mixture of Na_3_P and predominantly Na_2_P─PNa_2_.^[^ ^18^ ^]^ However, the addition of proton sources like *t*BuOH selectively converts such mixtures in highly valuable synthons like the PH_2_ˉ anion^[^ ^19^ ^]^ which in solubilized form has been extensively studied.^[^ ^20^ ^]^ Using Na/K alloy in dimethoxyethane, P_4_ can be reduced to P^3^ˉ at + 50 °C but it is unclear whether there is contamination with polyphosphide anions.^[^ ^21^ ^]^ The formation of P_2_^4^ˉ or even larger Zintl clusters like P_7_^3^ˉ is a way to avoid the very high concentration of negative charge on a single P atom and therefore P^3^ˉ formation is rarely selective.^[^ ^22^, ^23^, ^24^ ^]^ We showed^[^ ^24^ ^]^ that the reduction of P_4_ with a β‐diketiminate Mg^I^ complex introduced by Jones^[^ ^25^ ^]^ is much less selective than the most recently reported reduction of P_5_^−^ in Cp*Fe(P_5_)^[^ ^26^ ^]^ but can be controlled by increased ligand bulk. Herein, we report a relatively selective full reduction of P_4_ at room temperature and describe the isolation of a first hydrocarbon‐soluble *s*‐block metal complex of the P^3^ˉ anion.

Key to the facile reduction of P_4_ is the recently reported conversion of a sodium magnesyl complex (**V**, Scheme 2)^[^ ^27^ ^]^ to a redox‐active metal crown (**VI**).^[^ ^28^ ^]^ The latter is formally a Mg^0^ complex that consists of a magnesyl anion (BDI*)Mgˉ which is integrated in a ring of Na^+^ and N″ˉ ligands (N″ = N(SiMe_3_)_2_ and BDI* = HC[(*t*Bu)C═N‐DIPeP]_2_, DIPeP═2, 6‐CHEt_2_‐phenyl). Complex **VI** is able to reduce N_2_O by 2*e*ˉ transfer and subsequently captures the O^2^ˉ anion in a ring of metals (**VII**). The latter product is reminiscent of Mulvey's thoroughly investigated inverse crown ether complexes.^[^ ^29^ ^]^

**Scheme 2:**
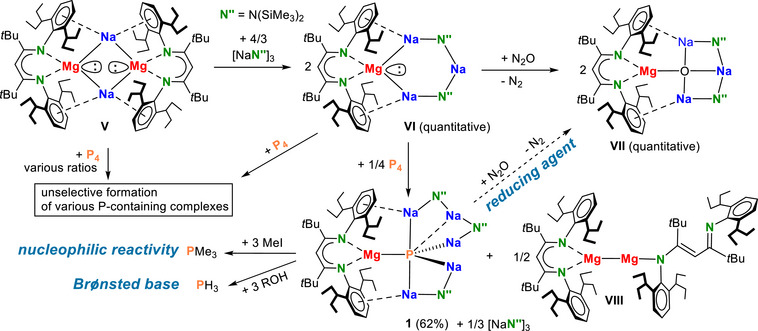
Synthesis of *s*‐block metal phosphide complex **1** and its diverse reactivity as a three‐fold nucleophile, base, or reducing agent.

Reduction of P_4_ with the original sodium magnesyl complex (**V**), which is a 4*e*ˉ donor, resulted besides the major products (BDI*)Na and [(BDI*)Mg]_2_ in the formation of many P_n_‐species (^1^H NMR: Figure S13; ^31^P NMR: Figure S14). Different educt ratios (**V**:P_4_ from 1:1 to 3:1) or reaction temperatures did not change this outcome.

We then focused on P_4_ reduction with the 2*e*ˉ donor **VI**. Our recently reported 2*e*ˉ reduction of N_2_ with an in situ prepared Ca^I^ reagent^[^ ^30^ ^]^ suggested that the metal crown complex may be able to stabilize the hitherto unknown isoelectronic P_2_^2^ˉ anion. Calculations show that reaction of **VI** with P_4_ is exothermic: **VI** + 0.5 P_4_→(BDI)MgNa_3_P_2_ (Δ*H* = −25.4 kcal mol^−1^; Figure S25). Unfortunately, reactions of **VI** with 0.5 equivalent of P_4_ resulted in a highly unselective conversion (Figures S17–S18). However, tuning the **VI**:P_4_ educt ratio led to a relatively selective formation of the P^3^ˉ complex (**1**, Scheme 2) and [(BDI*)Mg]_2_ (**VIII**); NMR of raw product Figures S1–S2. The **VI**:P_4_ ratio is critical. Too little reducing agent (**VI**:P_4_ = 1:0.5) resulted in incomplete P_4_ reduction and formation of a mixture of polyphosphorus anions. Too much reducing agent led to contamination of the products with educt **VI**. The correct stoichiometry (**VI**:P_4_ = 2:0.25) is rationalized by the equation: 2 **VI** + 0.25 P_4_→**1** + 0.5 **VIII** + 0.33 (NaN″)_3_. The full reduction of P to P^3^ˉ needs three electrons. Hence, one of the metal crowns (**VI**) reacts as a 2*e*ˉ‐donor and the second as sacrificial reagent that only transfers one electron and is the source of [Na‐N″‐Na]^+^. This explains the formation of Mg^I^ complex **VIII** and (NaN″)_3_ as side‐products (Figure S1). Complex **1** could be separated by crystallization in 62% yield.

The crystal structure of **1** (Figure 1a) shows that the six‐membered Mg‐Na‐N″‐Na‐N″‐Na ring of the metal crown broke into two arms of which the shorter end has been extended with a [Na‐N″‐Na^+^] cation, delivered by the second equivalent of **VI**. The terminal Na in the longer arm (Na3) is involved in anagostic interaction with a Et_2_CH‐substituent. Cleavage of the ring is the consequence of the odd 3– charge on the P^3^ˉ guest anion. However, like the metal crown in **VII**, the two arms effectively encapsulate the P^3^ˉ anion, stabilizing this first *s*‐block metal phosphido complex.

**Figure 1:**
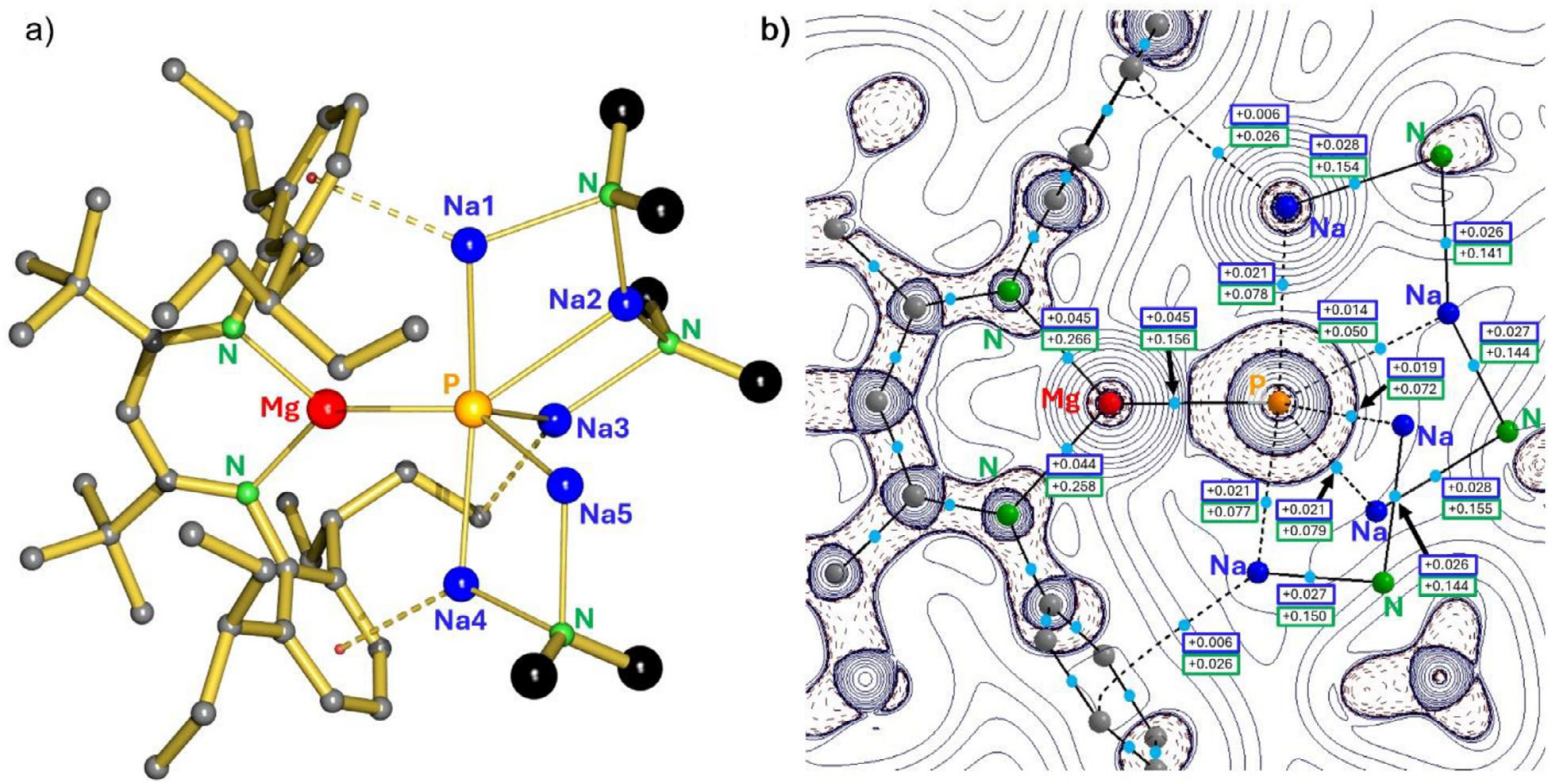
a) Crystal structure of the phosphido complex **1**; H atoms, and Me‐substituents of the N(SiMe_3_)_2_ ligands have been omitted for clarity. Selected bond distances are shown in Figure 2a. b) Atoms‐in‐molecules analysis of complex **1** showing a strong Mg─P bond path (solid line) and weaker Na─P bond paths (dashed lines) with bond‐critical‐points (bcp's) in light‐blue. The electron density *ρ*(r) and the Laplacian **∇** ^2^ *ρ*(r) in the bcp's are shown in a.u. in blue and green boxes, respectively.

Due to oxidation of the Mg^0^ centre, the Mg─Na bonds in **VI** are lost but the Ar···Na^+^ contacts are intact (average Ar_center_─Na in **1**: 2.727 Å; *cf*. in **VI**: 2.673 Å). The coordination geometry around the P^3^ˉ anion is close to trigonal bipyramidal with two Na^+^ cations in axial positions (Na1 and Na4) and a MgNa_2_ equatorial plane. The Na─P contacts vary from 2.745(1)–2.847(1) Å (average: 2.786 Å). However, there is an additional rather long Na2···P contact of 3.047(1) Å for which it is questionable whether this is still a bond or not. It is considerably longer than reported Na‐phosphide bonds, e.g. the Na─P bond of 2.824(2) Å in [(*μ_2_*‐Ph_2_P)Na]_∞_ but close to one of the Na─P contacts of 3.033(1) in dimeric [(*μ_2_*‐Ph_2_P)Na·PMDTA]_2_.^[^ ^31^ ^]^ Notably, all Na─P contacts in **1** are shorter than those in Na_3_P which span from 3.094(4) to 3.418(3) Å.^[^ ^17^ ^]^ Therefore, we consider the P─Na2 contact as a bonding interaction.

The Mg─P bond of 2.3850(8) Å is the shortest ever reported. It is considerably shorter than the Mg─P contacts in Mg_2_P_3_ (2.54–2.65 Å),^[^ ^32^ ^]^ the Mg─P bond in Mg[P(SiMe_3_)_2_]_2_·(THF)_2_ (2.5031(6) Å),^[^ ^31^ ^]^ or the Mg─PPh_2_ distance of 2.531(1) Å in a β‐diketiminate complex.^[^ ^17^ ^]^ It is also much shorter than the strong Mg─P bond in the phosphandiide complex [MgP(Si*t*Bu_3_)·(THF)]_4_ (average Mg─P: 2.572 Å) which has a cube‐like structure of alternating Mg^2+^ and (*t*Bu_3_Si)P^2^ˉ anions.^[^ ^33^ ^]^ The relatively long bonds to RP^2^ˉ can be explained by the lower 2– charge and its *μ_3_*‐bridging nature to three Mg^2+^ cations, whereas in **1** there is only one Mg─P bond. This indicates that the very short Mg─P contact in **1** is likely the main bonding interaction in the complex.

Although the open crown structure of **1** is unsymmetric, its ^1^H NMR spectrum indicates high symmetry. There is only one resonance for the *t*Bu‐groups and one signal for the benzylic proton on the Et_2_CH‐substituents. On average, this fits with a *C*_2_ *_v_*‐symmetric surrounding for the BDI* ligand. Although there are three chemically different N″ ligands, the spectrum only shows one large singlet for the Me_3_Si group, demonstrating high dynamics. The short Na‐N″‐Na arm is in exchange with the longer Na‐N″‐Na‐N″‐Na arm and also all N″ ligands change position (Figure 2b). In an attempt to compute a transition state for this exchange process, we located a minimum that represents the intermediate for exchange of the short and long arms (Figure 2c). In this structure, one of the N(SiMe_3_)_2_ ligands (N2) is bound over a triangular Na_3_ site. Although this is an unusual coordination mode for amido ligands, this intermediate is only slightly higher in energy than **1** (Δ*H* = +2.2 kcal mol^−1^, Δ*G* = +5.1 kcal mol^−1^), reflecting highly dynamic character.

**Figure 2:**
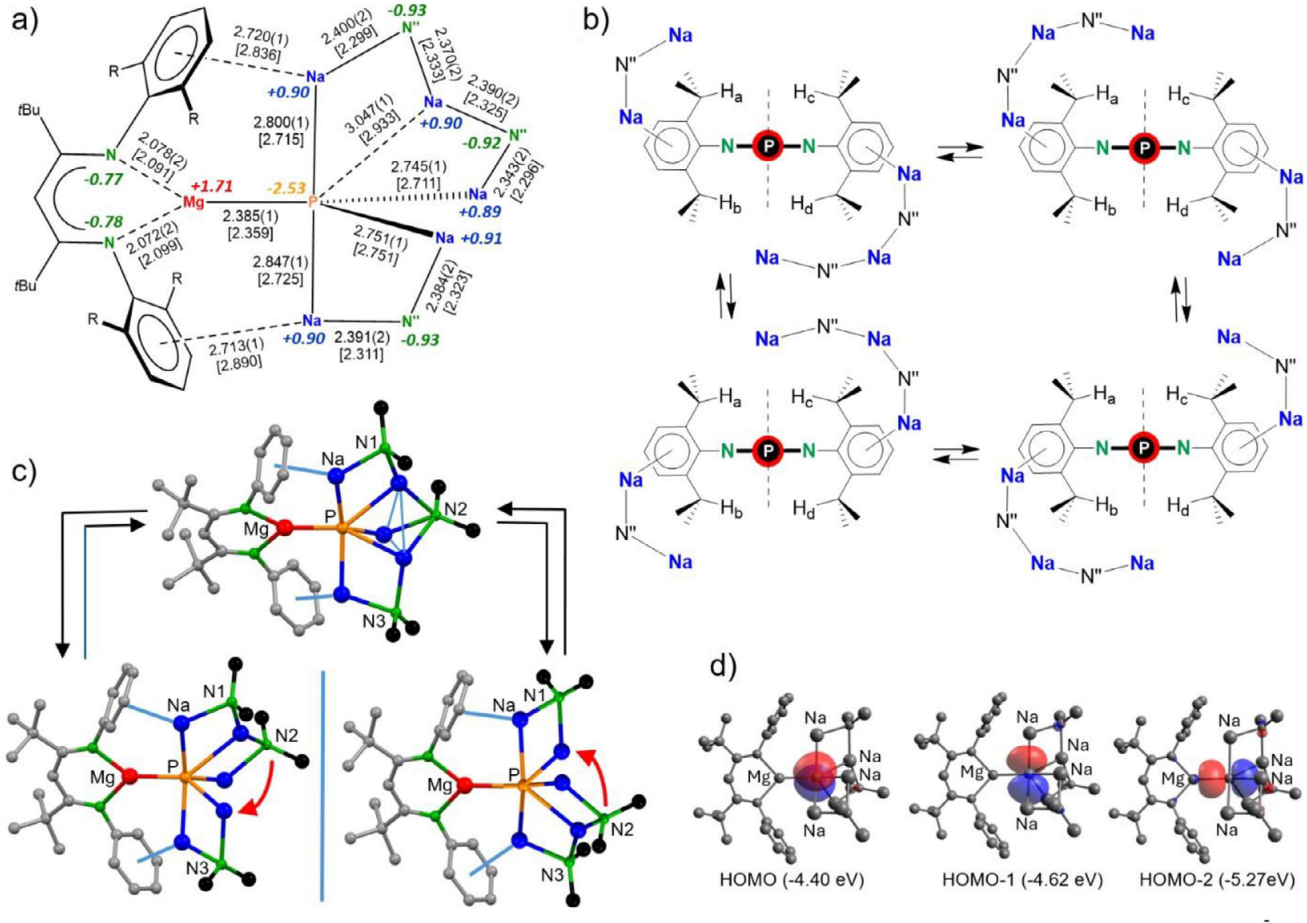
a) Comparison of experimental bond distances found in the crystal structure (Å) with calculated values [between squared brackets] and NPA charges for Mg, Na, N, and P atoms. b) Dynamics of the chiral complex **1** by exchange of short Na‐N″‐Na and long Na‐N″‐Na‐N″‐Na arms. c) Calculated structures for fast exchange between short and long arms in **1**. d) Selected molecular orbitals for **1**.

In contrast, the static crystal structure of **1** is highly unsymmetric and chiral. This asymmetry can be monitored by observing the benzylic CH groups which are situated in four different quadrants (Figure 2b). Cooling a methylcyclohexane‐*d*_14_ solution of **1** to −60 °C led to broadening of the benzylic resonance. At −80 °C decoalescence in four different signals is observed, as expected for a static structure (Figure S9). This is a good indication that the crystal structure is retained in solution. This was independently confirmed by a DOSY NMR experiment showing that all signals originate from one species (Figure S19).

Despite these dynamics, both arms are well able to stabilize the central P^3^ˉ anion, avoiding ligand scrambling and precipitation of insoluble Na_3_P or Mg_2_P_3_. Complex **1** is even at high temperatures up to +80 °C fully stable. The best description of **1** is therefore that of a tightly bound (BDI*)MgP^2^ˉ anion with chelating, stabilizing [Na_2_N″]^+^, and [Na_3_N″_2_]^+^ arms, reminiscent of pincer ligands. Latter arms are only loosely bound and in fast exchange.

A computational study at the B3PW91‐D3BJ/def2tzvp//B3PW91‐D3BJ/def2svp level of theory, which includes corrections for dispersion, shows that although the calculated values for the P─metal bonds are systematically slightly shorter than the experimental values, there is in general a good fit (Figure 2a). The formation of **1** is quite exothermic: 2 **VI** + 0.25 P_4_→**1** + 0.5 **VIII** + 0.33 (NaN″)_3_ (Δ*H* = −40.5 kcal mol^−1^, Δ*G*_298K_ = −30.1 kcal mol^−1^). Since P_4_‐to‐P^3‐^ breakdown needs at least a total of 12 electrons (6 x **VI**) and dynamic ligand and metal exchange between species, the exact computation of a possible mechanism is not realistic, and we did not attempt to fully model the reaction. However, we calculated the initial formation and encapsulation of a P_4_^2−^ anion with a typical butterfly structure (Figure S27). The size of the anion required an extension of the MgNa_3_‐cycle to a MgNa_4_‐cycle. Consistent with the highly stabilizing effect of the metal‐cycle, this reaction is highly exothermic (Δ*H* = −81.6 kcal mol^−1^, Δ*G*_298K_ = −62.7 kcal mol^−1^).

Natural‐population‐analysis (NPA) shows that bonding in **1** is mainly ionic (Figure 2a). There are high positive charges on Mg (+1.71) and Na (+0.89/+0.91) and a very negative charge of −2.53 on P; Atoms‐in‐molecules (AIM) calculated a charge of −2.15. Both are in agreement with ionic Mg^2+^─P^3^ˉ─Na^+^ bonding. For comparison, we also calculated the charge on the central P atom in Y phosphide complex **IV** ^[^ ^16^ ^]^ (NPA: −1.25, AIM: −1.73, Figure S26) which is considerably less ionic than complex **1**. However, bonding in **1** is less ionic than that in the metal crown oxide complex **VII** for which more extreme NPA charges were found (Mg: +1.81, Na: +0.93, O: −1.85).^[^ ^28^ ^]^ This shows that there is a slight electron transfer from the very electron‐rich P^3^ˉ anion to the metal cations, especially Mg^2+^. This is in line with the charge distribution and semi‐conducting nature of Mg_2_P_3_^[^ ^34^ ^]^ and Na_3_P.^[^ ^17^ ^]^ The band gap in the latter is much smaller than that in Li_3_P which is an isolator. This has been explained by a more efficient overlap of Na and P orbitals.^[^ ^17^ ^]^

AIM shows strong polarization of the electron density around the P^3^ˉ anion towards Mg^2+^ (Figure 1b). This analysis also shows a prominent Mg─P bond path with relatively high values for the electron density *ρ*(**r**) and the Laplacian **∇** ^2^ *ρ*(**r**) at the bond‐critical‐point (bcp). The Na─P bond paths are much weaker featuring values that are only half of that for the Mg─P bond. Even weaker bond paths are observed for the Na···Ar interactions. As deduced already from crystal structure data and solution dynamics, the AIM analysis is in line with an electronic structure in which Mg─P bonding dominates and Na─P bonding is weak and fluxional.

Molecular orbital analysis shows that the three highest occupied molecular orbitals, HOMO, HOMO‐1, and HOMO‐2 are mainly situated on the P^3^ˉ anion (Figure 2d). These are essentially represented by the orthogonal 3*p_x_*, 3*p_y_*, and 3*p_z_* orbitals of P and show little metal contributions (Figure S24). The highest metal contribution is found for Mg in HOMO‐2 which is directed along the Mg─P axis (P: 67.7%, Mg: 12.2%) but coefficients on Na are negligible. Natural‐localized‐molecular‐orbital (NLMO) analysis is in line with a P nucleus with four lone‐pairs of electrons of which three are dominated by the P *p*‐orbitals and one is associated with the P *s*‐orbital (Table S2).

Complex **1** can be considered a hydrocarbon‐soluble form of Na_3_P. As we showed previously for molecular calcium hydride or Mg^0^ complexes, solubilization can substantially impact reactivity.^[^ ^27^, ^35^, ^36^ ^]^ The high electron density of the phosphido P^3^ˉ anion promises rich and diverse reactivity. Reaction with Me_3_SiOH gave hydrolysis to PH_3_ which could be identified by ^31^P NMR. Its nucleophilic nature was shown by reaction with excess MeI and identification of PMe_3_ by ^31^P NMR. Complex **1** is a 3*e*ˉ reducing agent, even enabling the challenging reduction of N_2_O. This resulted in the formation of N_2_, various unidentified P‐containing species and the very stable inverse metal crown ether complex **VII**. Combined, these reactions show its Brønsted base character, its three‐fold nucleophilicity and its strongly reducing nature.

In summary, contrasting with earlier reported P_4_ reduction chemistry, which generally results in larger P_m_^n^ˉ anions and is often unselective, reduction with the hydrocarbon‐soluble, redox‐active metal crown complex **VI** is selective and results in full breakdown to the highly charged P^3^ˉ anion. The key to this unique ionic *s*‐block metal complex is the encapsulation of the phosphido anion in a MgNa_5_ pocket. To encapsulate the odd‐charged P^3^ˉ anion, the cyclic metal ring is broken. However, the two‐armed pincer ligand also effectively stabilizes the phosphido complex. Despite highly dynamic behaviour of both arms, a methylcyclohexane solution of complex **1** is stable up to at least +80 °C. A supporting computational study confirms the ionic nature of the phosphido complex. The highly charged P^3^ˉ anion features three high‐lying *p*‐type lone‐pairs and a lower‐lying *s*‐type lone‐pair. Despite its full valence shell, it is highly reactive showing Brønsted basic, nucleophilic, or strongly reducing character. We currently focus on the full reduction of other pnictide elements and the isolation of ionic *s*‐block metal complexes of the resulting pnictide tri‐anions.

## Supporting Information

Experimental details, NMR spectra, crystallographic details including ORTEP and details for DFT calculations. Deposition Number 2467297 contains the supplementary crystallographic data for this paper. These data are provided free of charge by the joint Cambridge Crystallographic Data Centre and Fachinformationszentrum Karlsruhe Access Structures service.

## Conflict of Interests

The authors declare no conflict of interest.

## Supporting information



Supporting Information

## Data Availability

The data that support the findings of this study are available in the Supporting Information of this article.
