# Supplementary material for: Full P4 to P3− Reduction with a Redox‐Active Metal Crown Complex
Source: Angew Chem Int Ed Engl. 2025 Sep 1;64(42):e202515157. doi: 10.1002/anie.202515157 (PMC12518698; doi:10.1002/anie.202515157)
Supplement: Supplementary file 1 — Supporting Information [file ANIE-64-e202515157-s001.pdf]

Table of Contents

1. Materials and methods 2
2. Complex synthesis 3
3. Reactivity of (BDI*)$MgNa_5N''_3$(P) (1) as threefold base and nucleophile 3
4. Spectroscopic data 4
5. Diffusion measurements (DOSY) 16
6. Crystal structure determination 18
8. DFT calculations 21
9. References 37

1. Materials and methods

All experiments were conducted in dry glassware under an inert nitrogen atmosphere by applying standard Schlenk techniques or gloveboxes (MBraun) using freshly dried and degassed solvents. All solvents were degassed with nitrogen, dried over activated aluminum oxide (Innovative Technology, Pure Solv 400-4-MD, Solvent Purification System), and then stored under inert atmosphere over molecular sieves (3 Å) unless noted otherwise. Cylcohexane-d_{12} (Deutero GmbH) and C_6D_6 (Sigma Aldrich) were purchased as indicated and dried over molecular sieves (3 Å). The following compounds were purchased as indicated and used without further purification: NaN'' (N'' = $N(SiMe_3)_2$, 95%, Sigma Aldrich), $HOSiMe_3$ (≥97.5% (GC), Sigma Aldrich), MeI (purum, ≥99.0% (GC), Sigma Aldrich). The following compounds were synthesized or purified according to literature procedures: $[(BDI^*)MgNa]_2$,[1] $(BDI^*)MgNa_3N''_2$ (**V**).[2] (BDI* = β-diketiminate ligand: HC[C(*t*Bu)N-DIPeP]$_2$; DIPeP = 2,6-CH(Et)$_2$-phenyl), P_4.[3]

NMR spectra were measured on Bruker Avance III HD 400 MHz and Bruker Avance III HD 600 MHz spectrometers. Chemical shifts (δ) are denoted in ppm (parts per million), coupling constants in Hz (Hertz). For describing signal multiplicities common abbreviations are used: s (singlet), t (triplet), m (multiplet) and br (broad). Spectra were referenced to the solvent residual signal. Assignments of resonance signals in the ^1H and $^{13}C\{^1H\}$/^{13}C-APT NMR spectra were made based on two-dimensional NMR correlation (HSQC, HMBC, COSY) experiments. Elemental analysis was performed with an Hekatech Eurovector EA3000 analyzer. All crystal structures have been measured on a SuperNova (Agilent) diffractometer with dual Cu and Mo microfocus sources and an Atlas S2 detector.

Crystallographic data have been deposited with the Cambridge Crystallographic Data Centre as supplementary publication numbers: CCDC 2467297

2. Complex synthesis

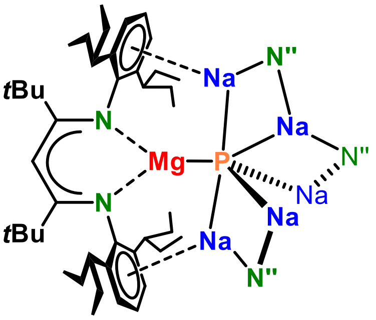

Synthesis of (BDI*)MgNa$_5$N’’$_3$(P) (1)

(BDI*)MgNa$_3$N$_2$’’ was prepared *in situ* by suspending [(BDI*)MgNa]$_2$ (60.0 mg, 45.4 μmol, 3.5 equiv.) and (NaN’’)$_3$ (33.2, 60.3 μmol, 4.7 equiv.) in hexanes (3 ml) and stirring it for 10 min at room temperature. The quantitative formation was verified by ^{1}H-NMR spectroscopy. P$_4$ (1.6 mg, 12.9 μmol, 1.0 equiv.) was added to the dark red solution and the mixture was stirred at room temperature for 19 h. The dark red colour changed to light red and a colourless precipitate of (NaN‘‘)$_3$ formed. All solids were filtered off and discarded. The filtrate was dried *in vacuo* and the obtained solid was redissolved in pentane (500 µl). The obtained solution was stored at –30 °C. After 3 days (BDI*)MgNa$_5$N’’$_3$(P) (**1**) (40.2 mg, 31.8 μmol, **62%**) was obtained as bright orange crystals, which were suitable for single crystal X-ray diffraction.

^{1}H-NMR: (600 MHz, C$_6$D$_{12}$, 25°C): δ = 7.19-7.14 m, (6H, *meta*/*para*-CH$_{arom}$), 5.43 (s, 1H, CH-backbone), 2.98-2.93 (m, 4H, C**H**Et$_2$), 2.20-2.12 (m, 4H, CH(C**H$_2$**CH$_3$)$_2$), 2.10-2.03 (m, 4H, CH(C**H$_2$**CH$_3$)$_2$), 1.98-1.91 (m, 4H, CH(C**H$_2$**CH$_3$)$_2$), 1.71-1.64 (m, 4H, CH(C**H$_2$**CH$_3$)$_2$), 1.18 (s, 18H, *t*Bu), 1.07 (t, $^3J_{H,H}$ = 7.3 Hz, 12H, CH(CH$_2$C**H$_3$**)$_2$), 0.96 (t, $^3J_{H,H}$ = 7.4 Hz 12H, CH(C**H$_2$**CH$_3$)$_2$), -0.07 (s, 54H, SiMe$_3$) ppm.

^{13}C{^{1}H}-NMR: (151 MHz, C$_6$D$_{12}$ 25°C): δ = 176.7 (2C, CN-backbone), 151.2 (2C, N-**C**$_{arom}$), 141.4 (4C, *ortho*-C$_{arom}$), 126.0 (4C, *meta*-C$_{arom}$), 123.6 (2C, *para*-C$_{arom}$), 97.2 (1C, CH-backbone), 45.0 (2C, **C**Me$_3$ *t*Bu), 41.5 (4C, **C**HEt$_2$), 33.5 (6C, CH$_3$ *t*Bu), 28.2, 25.1 (two signals: 4C, CH(**C**H$_2$CH$_3$)$_2$), 12.0, 11.9 (two signals; 4C, CH(CH$_2$**C**H$_3$)$_2$), 7.2 (18C, SiMe$_3$) ppm.

^{31}P{^{1}H}-NMR: (243 MHz, C$_6$D$_{12}$, 25°C): δ = – 426.9 (s, 1P, P$^{3–}$) ppm.

Elemental analysis for C$_{61}$H$_{123}$MgN$_5$Na$_5$PSi$_6$ (M = 1265.43 g/mol): Calc.: C 57.90, H 9.80, N 5.53 %. Found: C 58.45, H 9.52, N 5.87 %. Although the C value is outside the range viewed as establishing analytical purity, it is provided to illustrate the best values obtained to date.

3. Reactivity of (BDI*)MgNa$_5$N’’$_3$(P) (1) as threefold base and nucleophile

(BDI*)MgNa$_5$N’’$_3$(P) (1) with HOSiMe$_3$

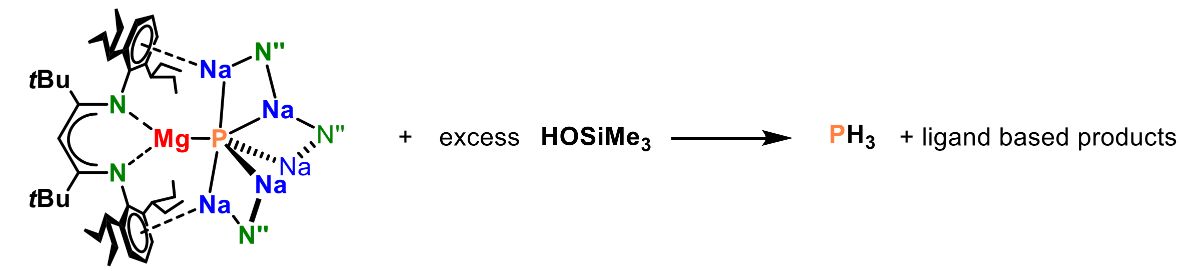

5 mg of (BDI*)MgNa$_5$N''$_3$(P) (**1**) were dissolved in C_6D_{12} and a drop of $HOSiMe_3$ was added. The orange solution instantaneousely turned yellow, indicating full conversion of **1**.

^{31}P-NMR spectroscopy revealed the formation of PH_3 demonstrating the threefold basicity of the P^{3-} core (**Figure S11**).

(BDI*)MgNa$_5$N''$_3$(P) (1) with MeI

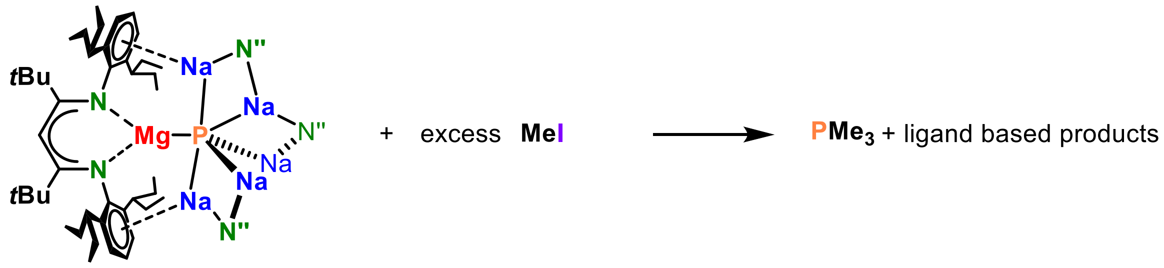

5 mg of (BDI*)MgNa$_5$N''$_3$(P) (**1**) were dissolved in C_6D_{12} and a drop of MeI was added. The orange solution instantaneousely turned yellow, indicating full conversion of **1**.

^{31}P-NMR spectroscopy revealed, besides ligand based products, the formation of PMe_3 proofing the threefold nucleophilicity of the P^{3-}-core (**Figure S12**).

4. Spectroscopic data

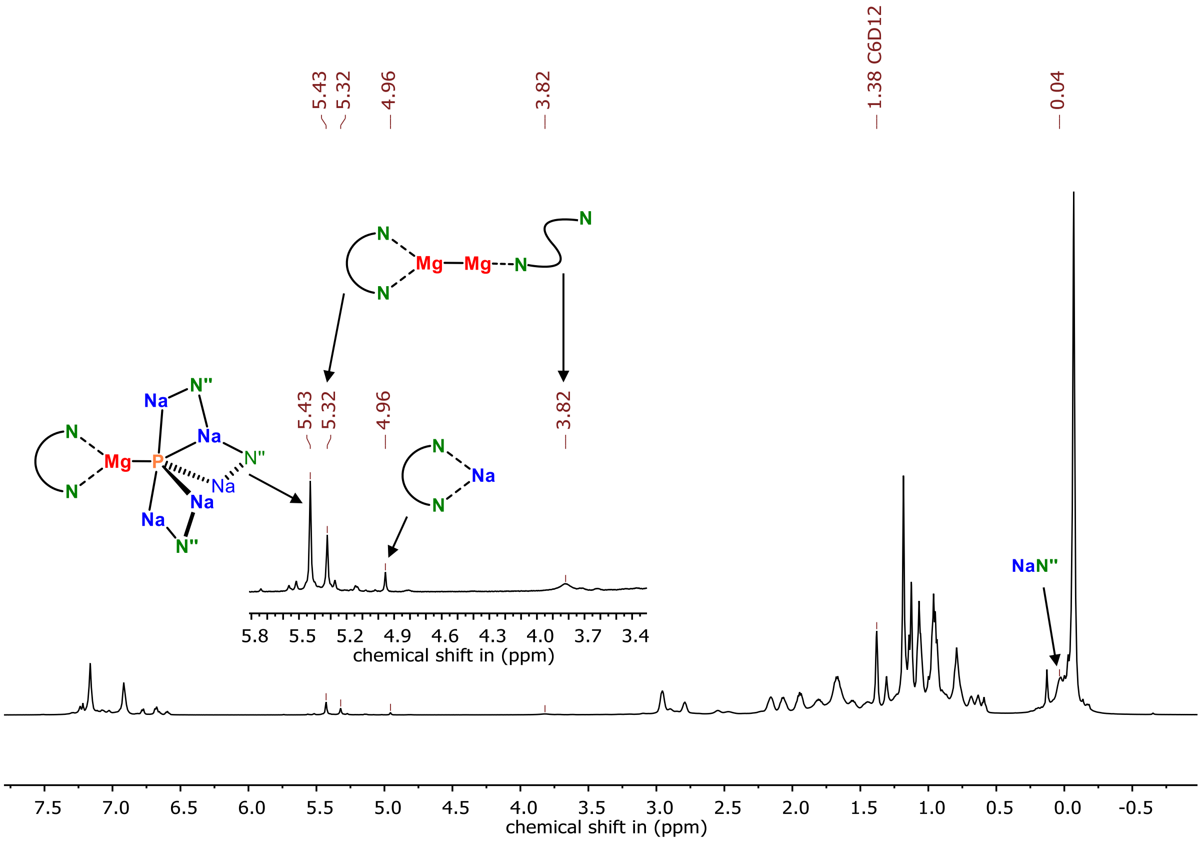

Figure S1. ^{1}H NMR (600 MHz, 298 K, cyclohexane-d_{12}) spectrum of the crude reaction mixture of the reaction of 2 equiv. (BDI*)MgNa$_3$N''$_2$ (**V**) with 0.25 equiv. P_4. (BDI*)MgNa$_5$N''$_3$(P) (**1**) is formed as a major species, alongside [(BDI*)Mg]$_2$ (**VII**). Only small quantities of free NaN'' are detected, due to bad solubility of this amide in cyclohexane and its precipitation during the reaction. Due to the side reaction of **1** with P_4 (BDI*)Na and other minor product peaks are visible.

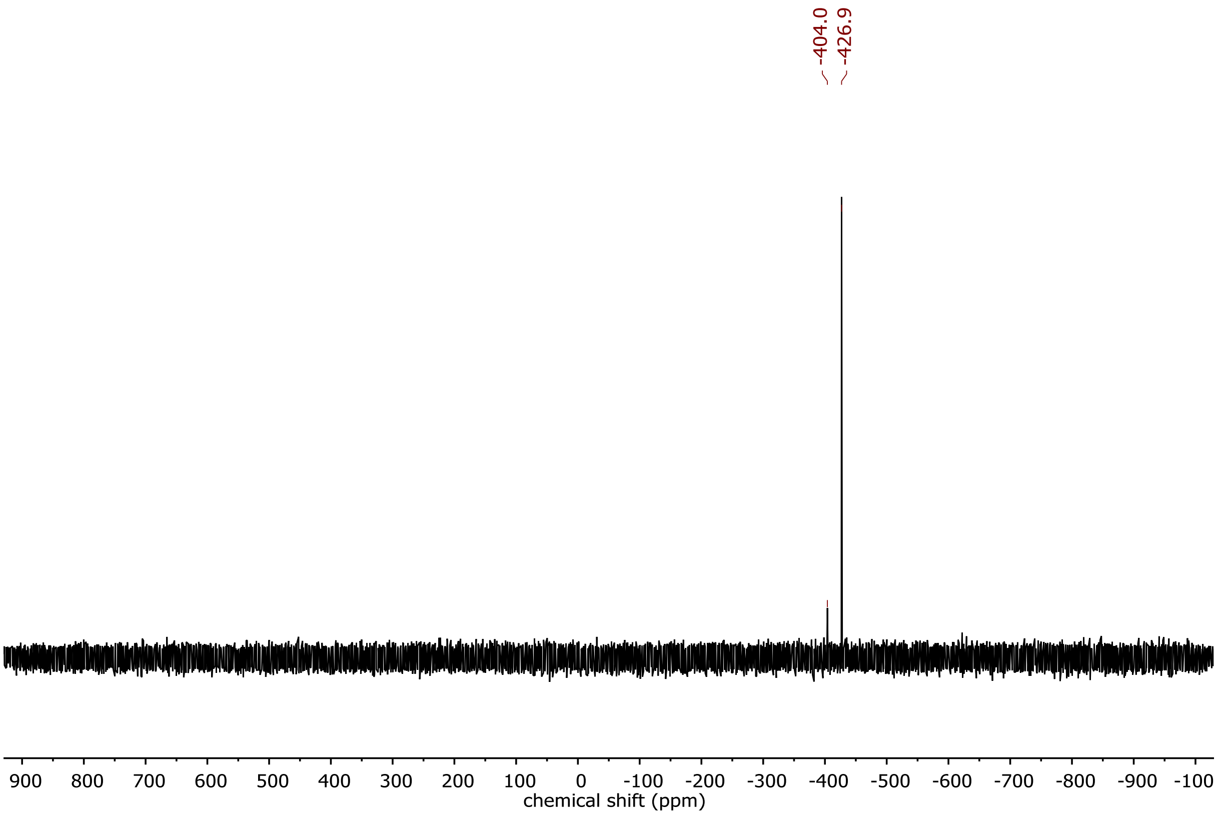

Figure S2. ^{31}P NMR (243 MHz, 298 K, cyclohexane-d_{12}) spectrum of the crude reaction mixture of the reaction of (BDI*)$MgNa_3N''_2$ (**V**) with 0.25 eq. P_4. (BDI*)$MgNa_5N''_3$(P) (**1**) is formed as a major species, alongside one minor species at –404.0 ppm

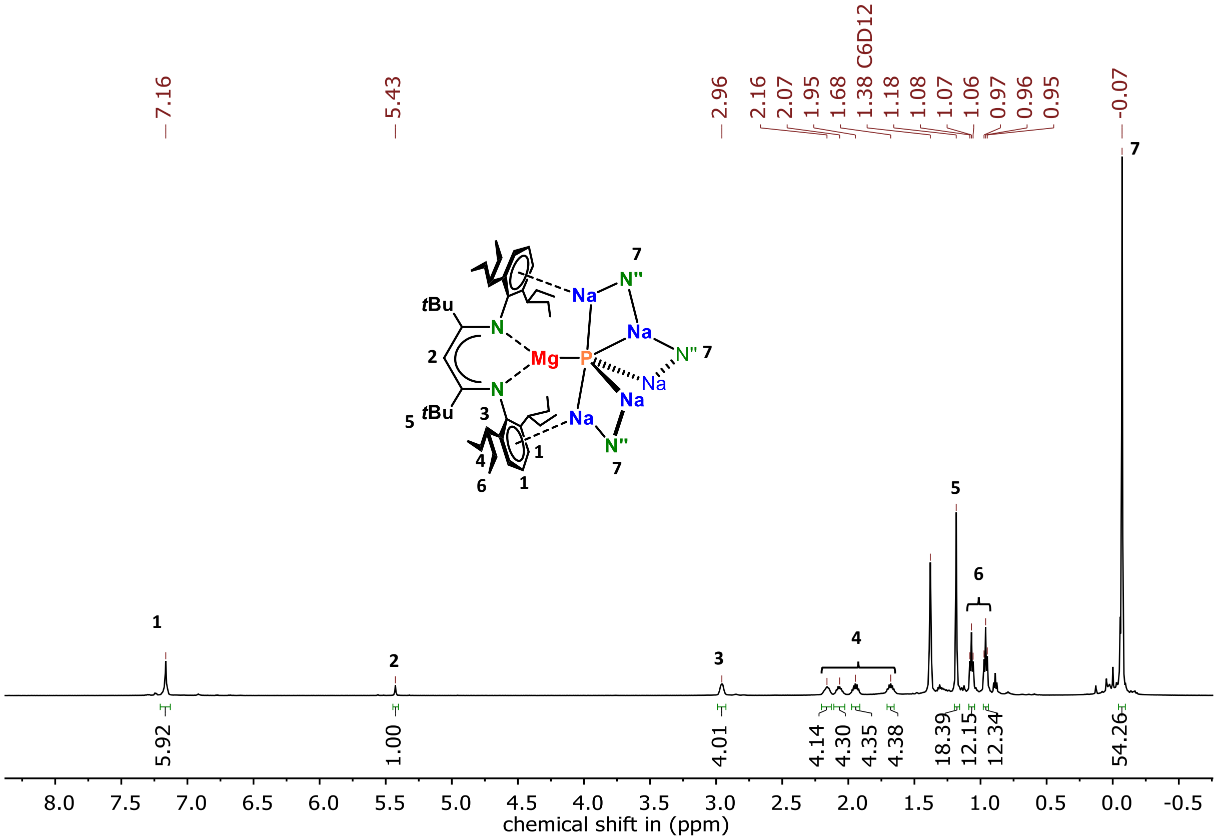

Figure S3. ^{1}H NMR (600 MHz, 298 K, cyclohexane-d_{12}) spectrum of (BDI*)$MgNa_5N''_3(P)$ (**1**).

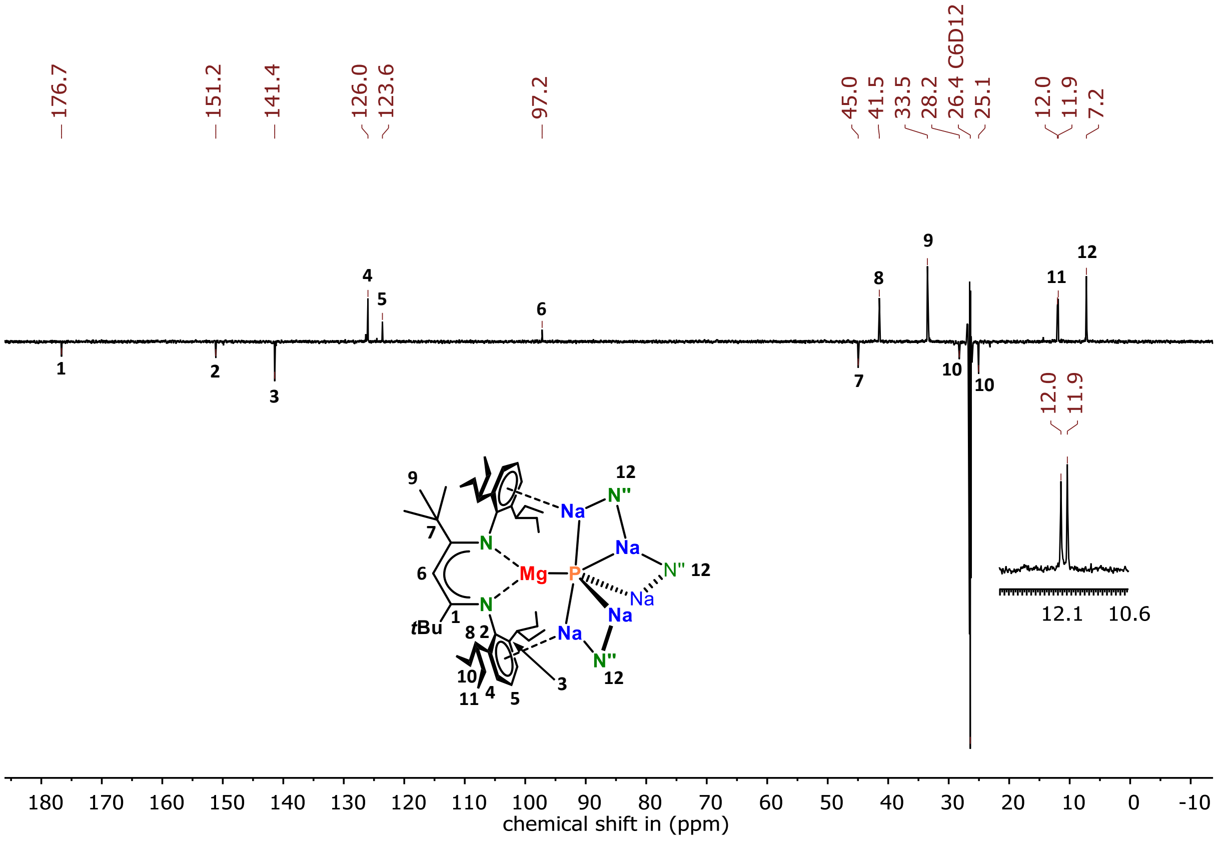

Figure S4. ^{13}C-APT NMR (151 MHz, 298 K, cyclohexane-d_{12}) spectrum of (BDI*)$MgNa_5N''_3(P)$ (**1**).

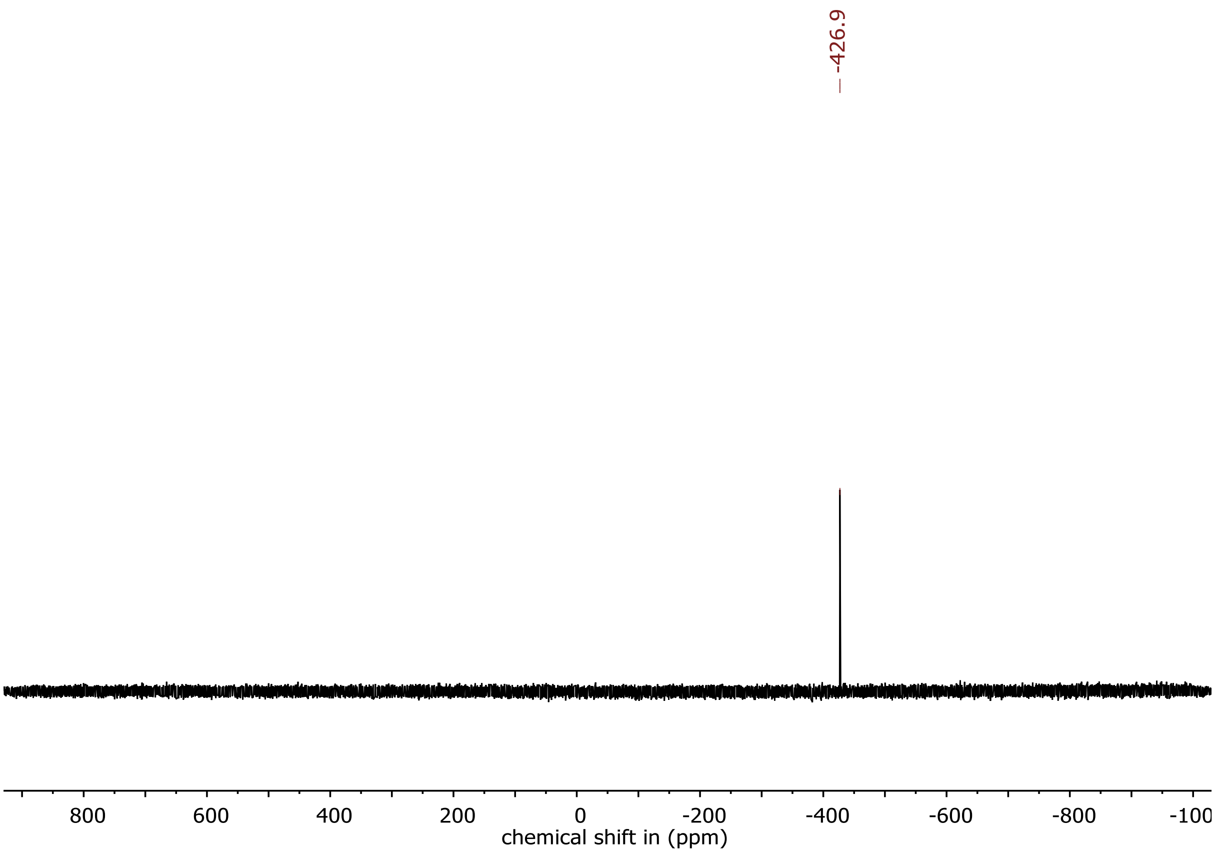

Figure S5. $^{31}P\{^{1}H\}$ NMR (243 MHz, 298 K, cyclohexane-d_{12}) spectrum of (BDI*)MgNa$_5$N’’$_3$(P) (**1**).

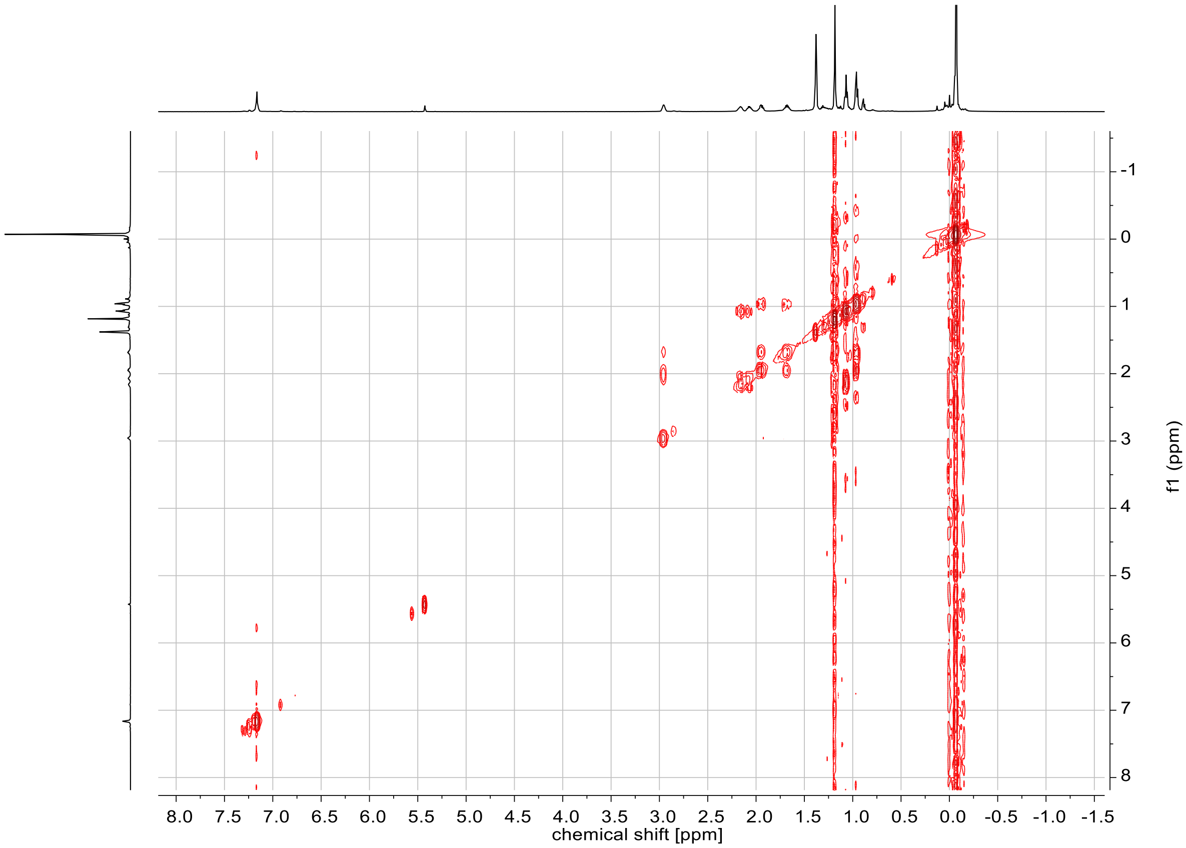

Figure S6. 2D-COSY NMR (600 MHz, 298 K, cyclohexane-d_{12}) spectrum of (BDI*)MgNa$_5$N’’$_3$(P) (**1**).

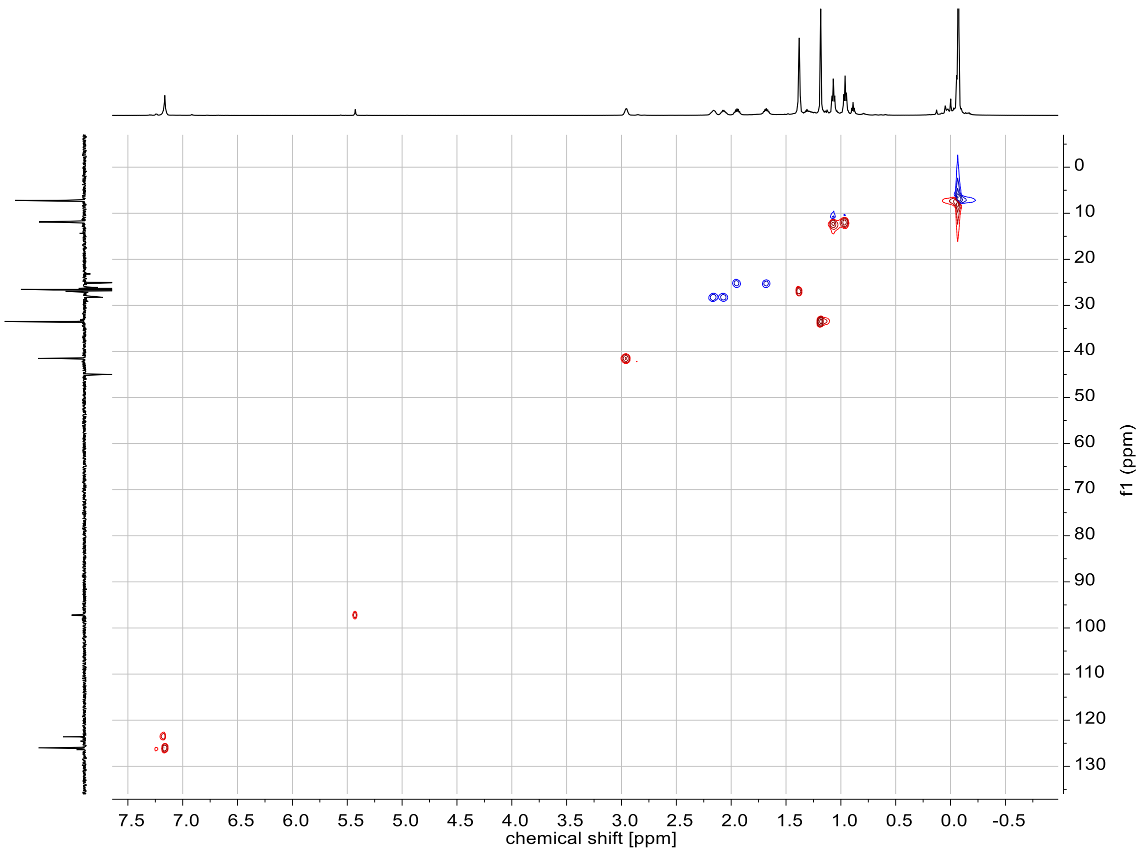

Figure S7. 2D-HSQC NMR spectrum (600/151 MHz, 298 K, cyclohexane-d_{12}) of (BDI*)MgNa$_5$N’’$_3$(P) (**1**).

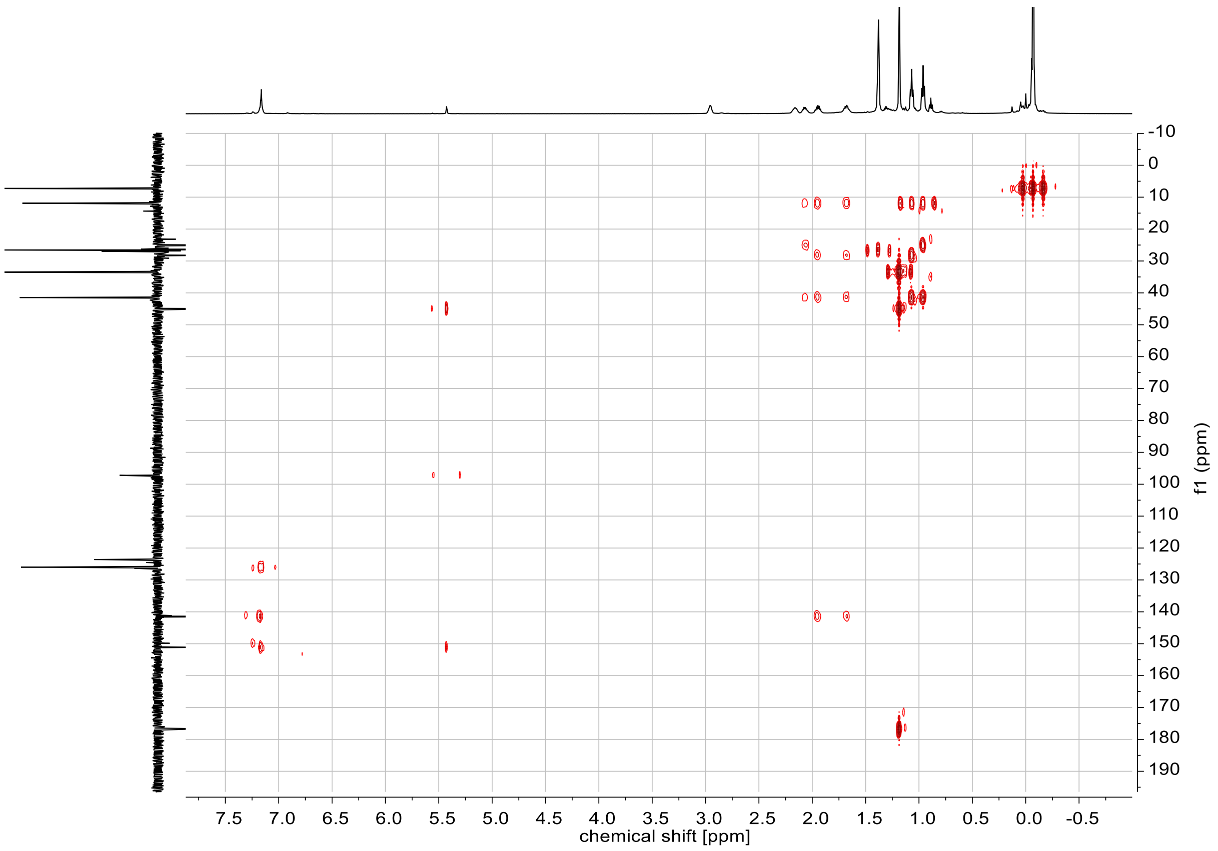

Figure S8. 2D-HMBC NMR spectrum (600/151 MHz, 298 K, cyclohexane-d_{12}) of (BDI*)MgNa$_5$N’’$_3$(P) (**1**).

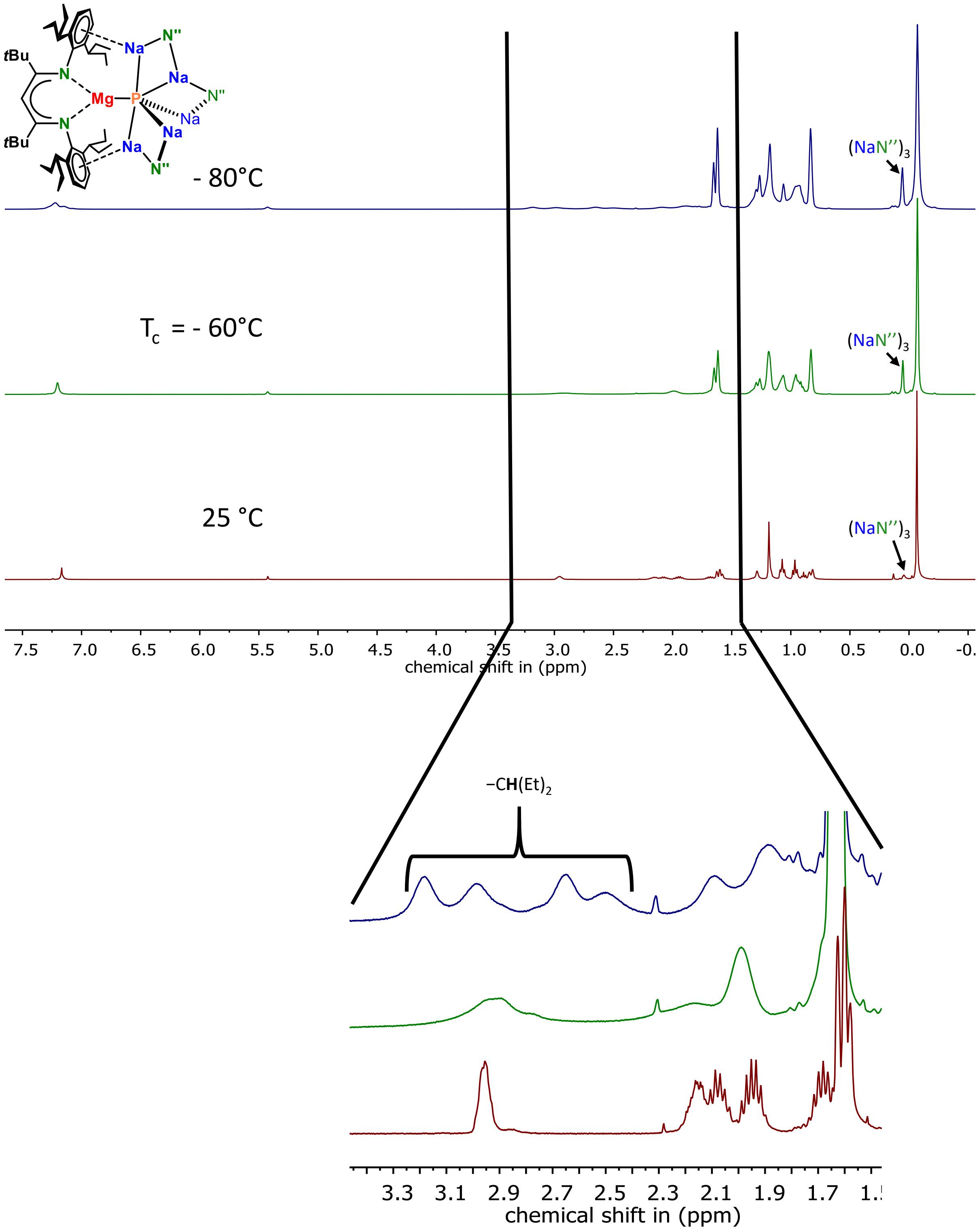

Figure S9. Temperature dependent ^{1}H NMR spectra (400.15 MHz, C_7D_{14}) of (BDI*)MgNa$_5$N''$_3$(P) (**1**). The benzylic C**H**(Et)$_2$ protons split into four signals.

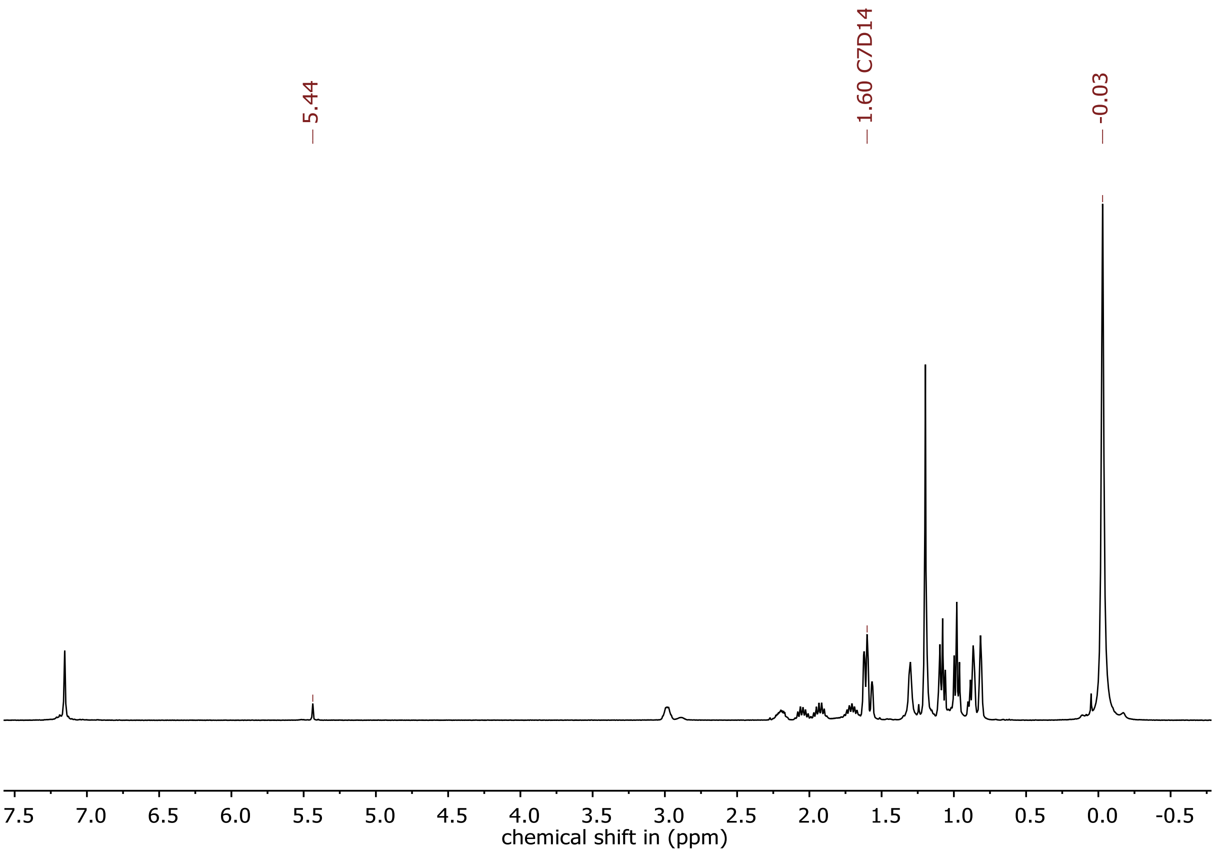

Figure S10. ^{1}H NMR spectrum (400.15 MHz, 363 K, C_7D_{14}) of (BDI*)$MgNa_5N''_3$(P) (**1**). The complex is stable up to +90 °C.

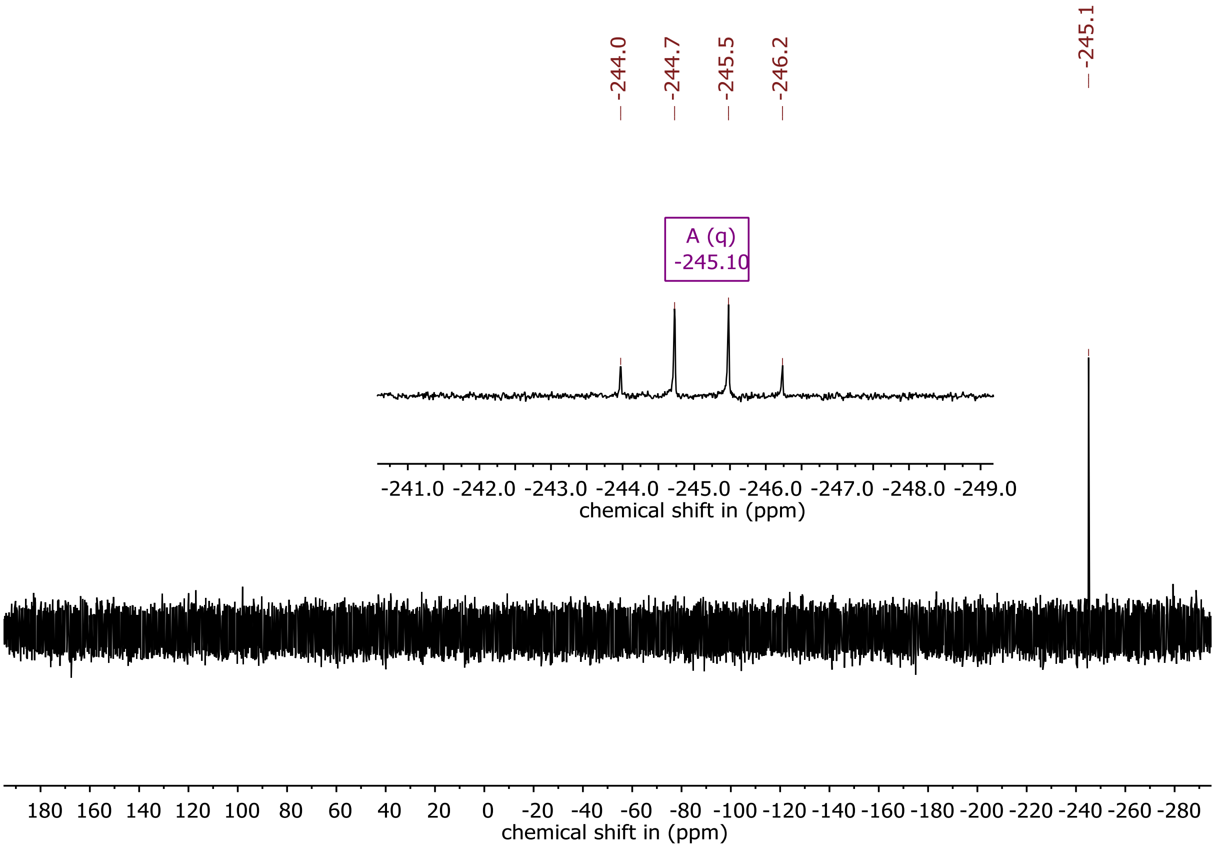

Figure S11. ^{31}P NMR (243 MHz, 298 K, cyclohexane-d_{12}) spectrum of the reaction of (BDI*)$MgNa_5N''_3$(P) (**1**) with $HOSiMe_3$.The triple protonation of P^{3-} towards PH_3 is proven by the multiplicity (quartet), the coupling constant ($^1J_{H,P}$ = 183 Hz) and the chemical shift of the signal at δ= -245.1 ppm.

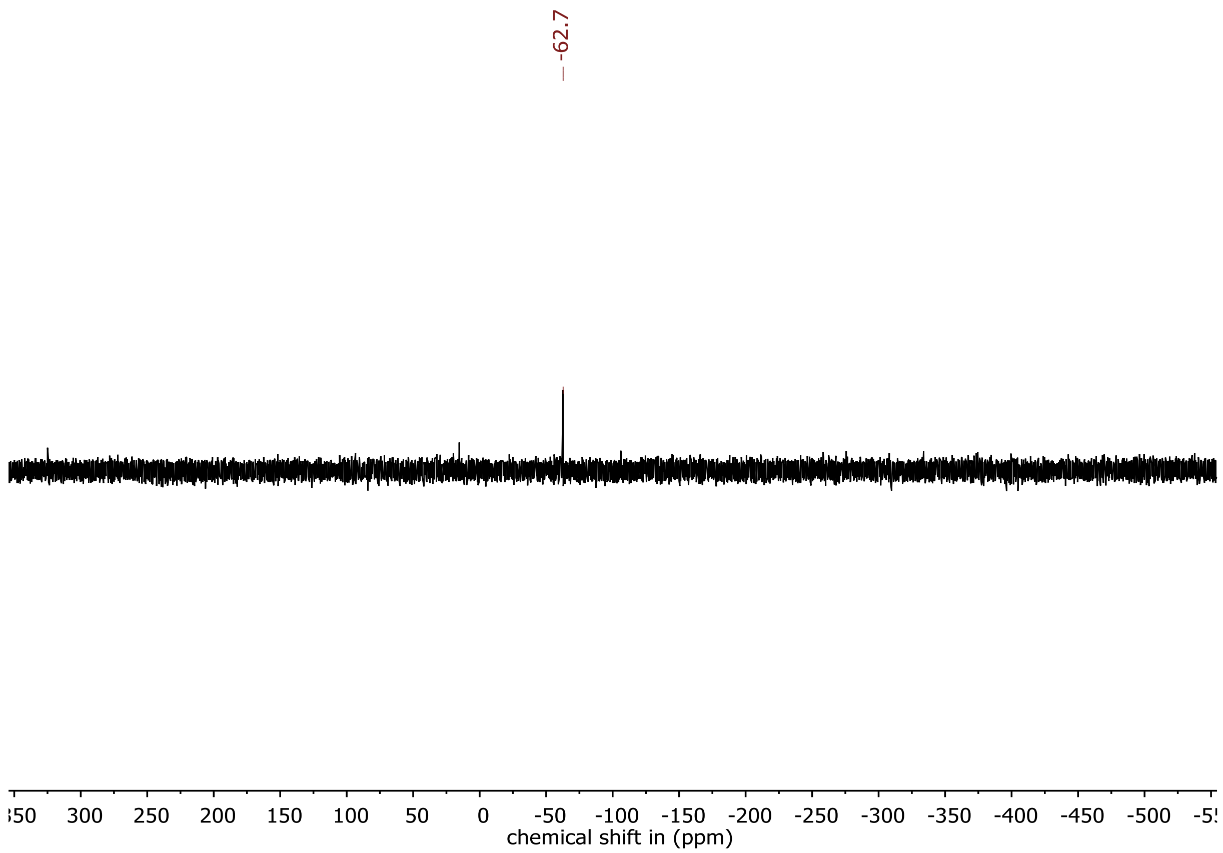

Figure S12. ^{31}P NMR (243 MHz, 298 K, cyclohexane-d_{12}) spectrum of the reaction of (BDI*)MgNa$_5$N''$_3$(P) (**1**) with MeI.The triple nucleophilic attack of P^{3-}at MeI towards PMe_3 is proven by the multiplicity (singlet) and the chemical shift of the signal at δ = -62.7 ppm.

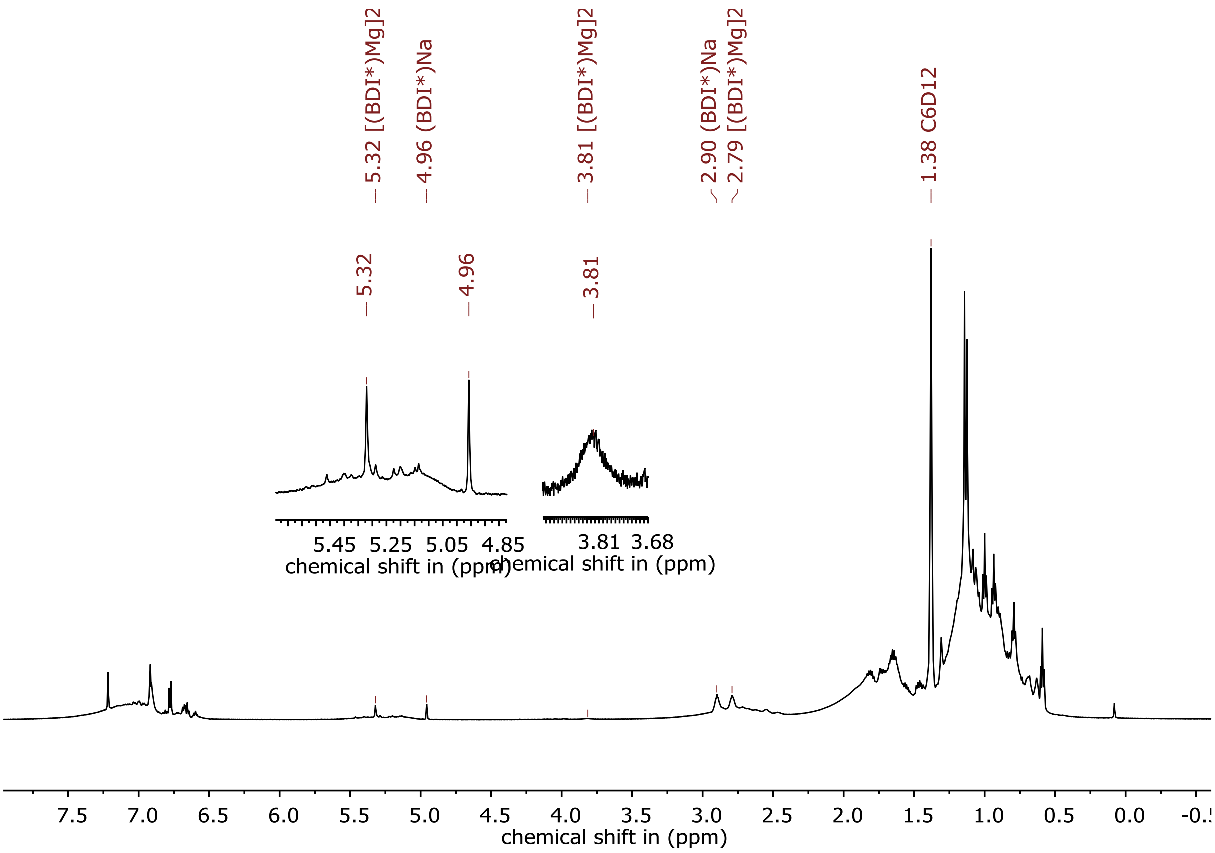

Figure S13. ^{1}H NMR (600 MHz, 298 K, cyclohexane-d_{12}) spectrum of the reaction of $[(BDI^{*})MgNa]_2$ (**IV**) with P_4. $[(BDI^{*})Mg]_2$ and $(BDI^{*})Na$ are formed as major products.

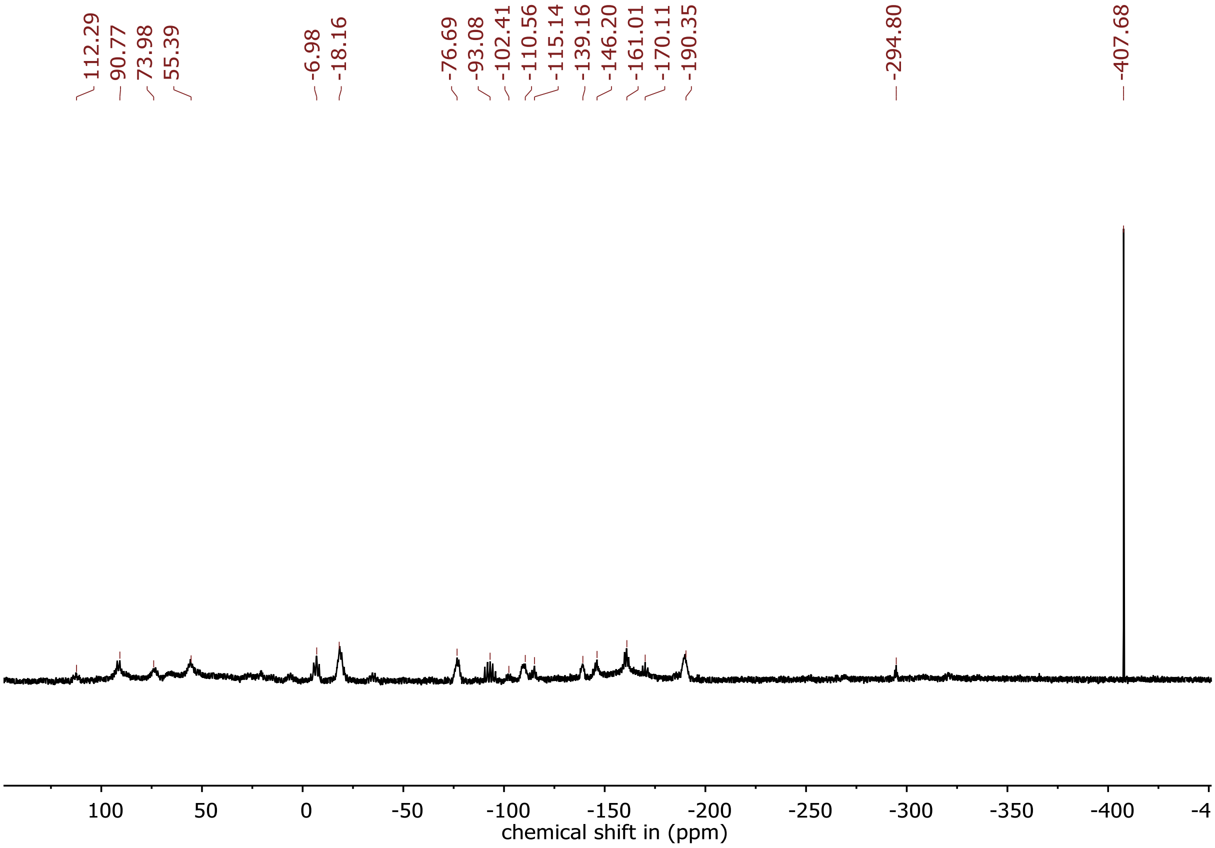

Figure S14. $^{31}P\{^{1}H\}$ NMR (243 MHz, 298 K, cyclohexane-d_{12}) spectrum of the reaction of $[(BDI^{*})MgNa]_2$ (**IV**) with P_4. The presence of multiplets suggests cluster formation of phosphorous.

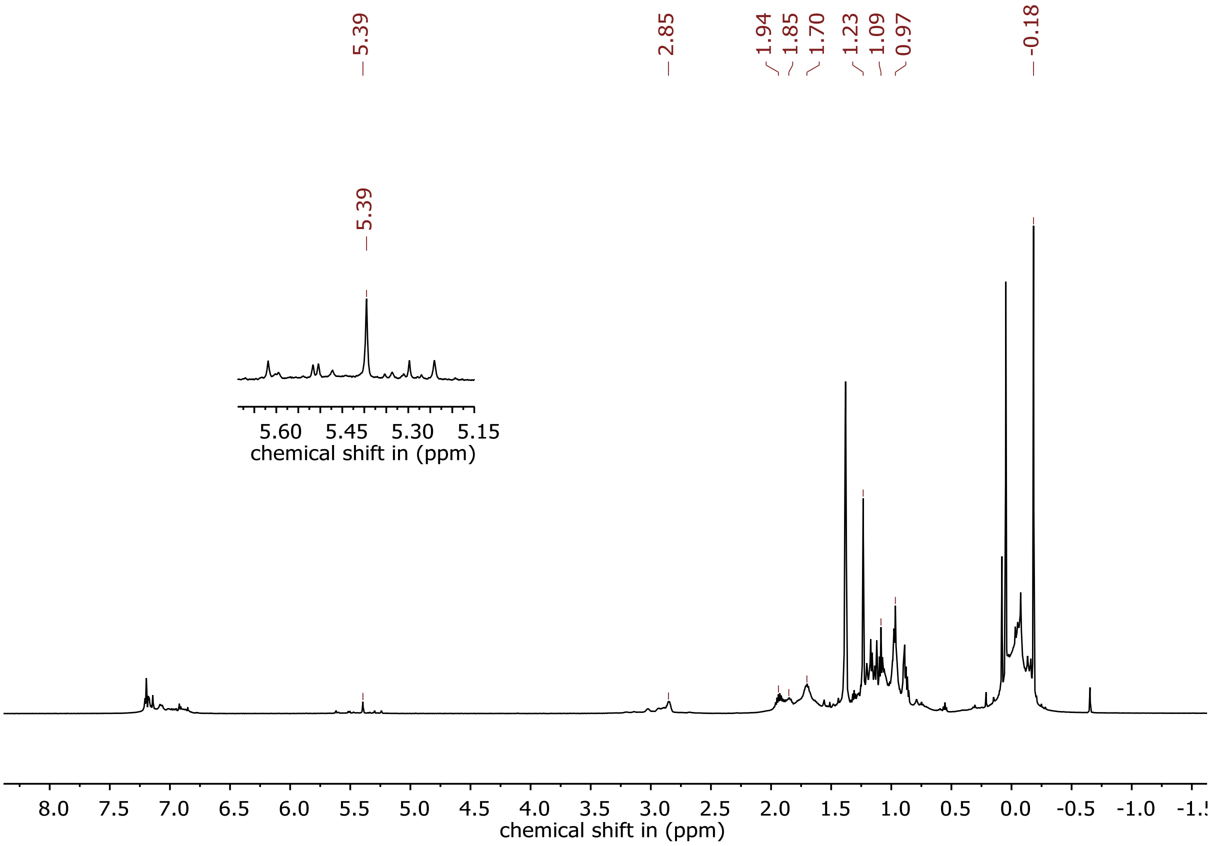

Figure S15. ^{1}H NMR (600 MHz, 298 K, cyclohexane-*d*$_{12}$) spectrum of the reduction of N_2O with (BDI*)MgNa$_5$N''$_3$(P) (**1**). Besides other unidentified products, the inverse crown complex (BDI*)MgNa$_3$N''$_2$(O) (**VI**) is formed.

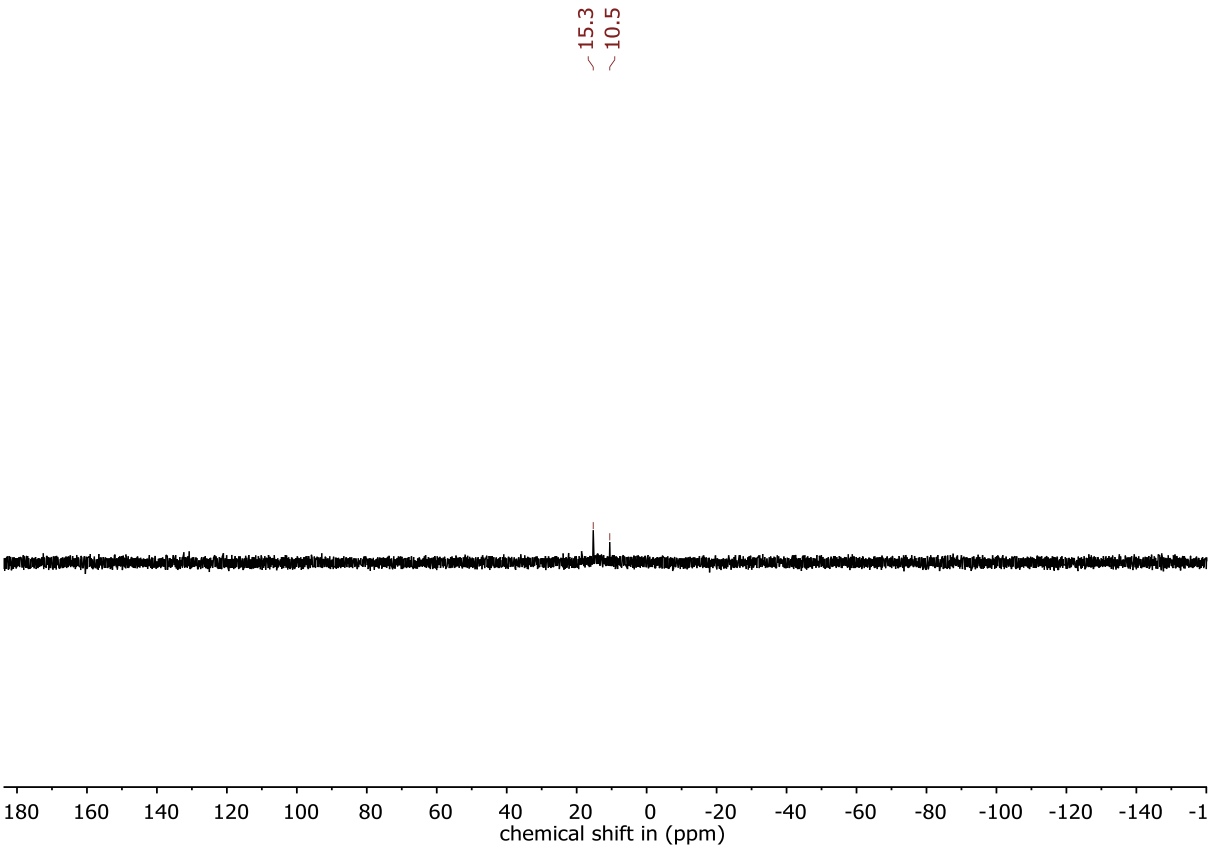

Figure S16. $^{31}P\{^{1}H\}$NMR (243 MHz, 298 K, cyclohexane-*d*$_{12}$) spectrum of the reduction of N_2O with (BDI*)MgNa$_5$N''$_3$(P) (**1**).

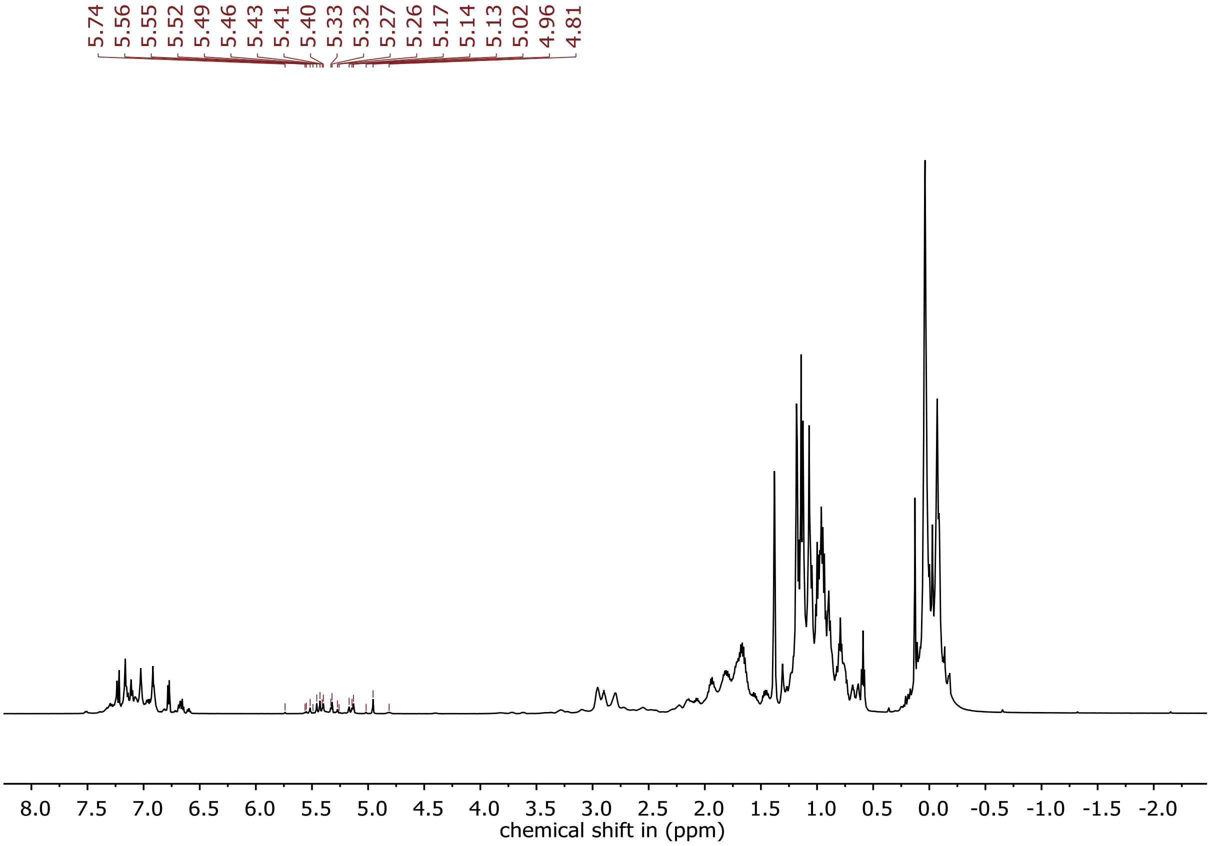

Figure S17. ^{1}H NMR (600 MHz, 298 K, cyclohexane-d_{12}) spectrum of the reaction of (BDI*)$MgNa_3N''_2$(**V**) with 0.5 equivalents P_4.

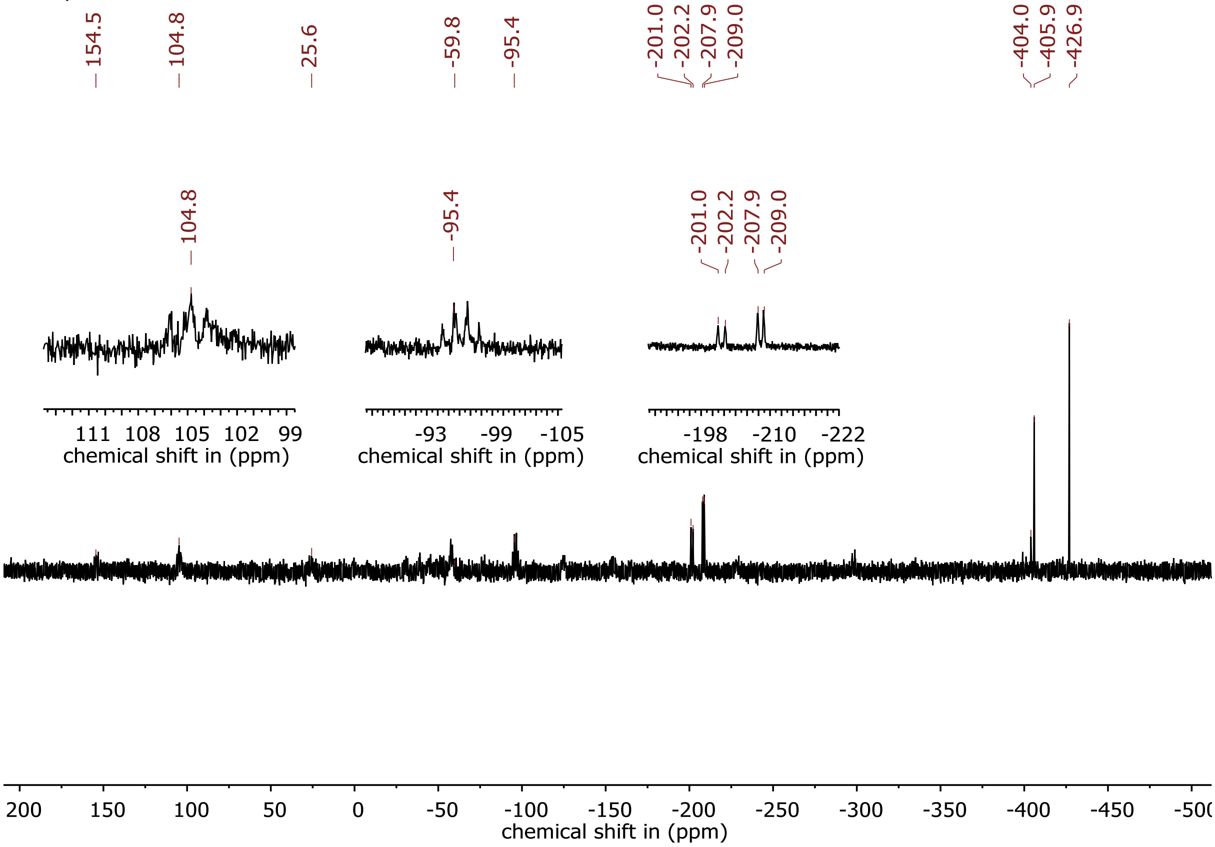

Figure S18. $^{31}P\{^{1}H\}$NMR (243 MHz, 298 K, cyclohexane-d_{12}) spectrum of the reaction of (BDI*)$MgNa_3N''_2$ (**V**) with 0.5 equivalents P_4. The presence of multiplets suggests cluster formation of phosphorous.

5. Diffusion measurements (DOSY)

Diffusion measurements were conducted on a Bruker AVANCE NMR spectrometer operating at 600.13 MHz for proton resonance equipped with a 5 mm PABDO BB/19F-1H/D probe with Z-GRD and actively shielded gradient coil with a maximum gradient strength of 5.3500094 G/mm (at 10 A).

Parameter optimization was carried out empirically employing the pulse program ledbpgp2s1D using stimulated echo and LED (D21 = 5 ms, longitudinal eddy current delay as a Z-filter) with bipolar gradient pulses (P30) and two spoiling gradients (P19 = 600 µs) leading to values for gradient pulse length (P30 = 1900 [µs], in case of bipolar gradients little DELTA*0.5) and diffusion time (D20 = 60 ms, big DELTA). Delay for gradient recovery was set to 200 µs.

The diffusion experiment was executed with variable gradients from 2% to 98% gradient strength with 32 increment values (difframp calculated with the AU-program DOSY). In this case the pulse program ledbpgp2s was applied for data aquiring of this pseudo-2D Experiment. Data processing was performed with the T1/T2 software package (SimFit) of TopSpin (version 3.2, Bruker Biospin) by fitting area data (integration of all peaks of interest of the same molecule) of diffusion decays. From these Stejskal-Tanner fitting curves calculated diffusion constants were obtained and assimilated statistically.

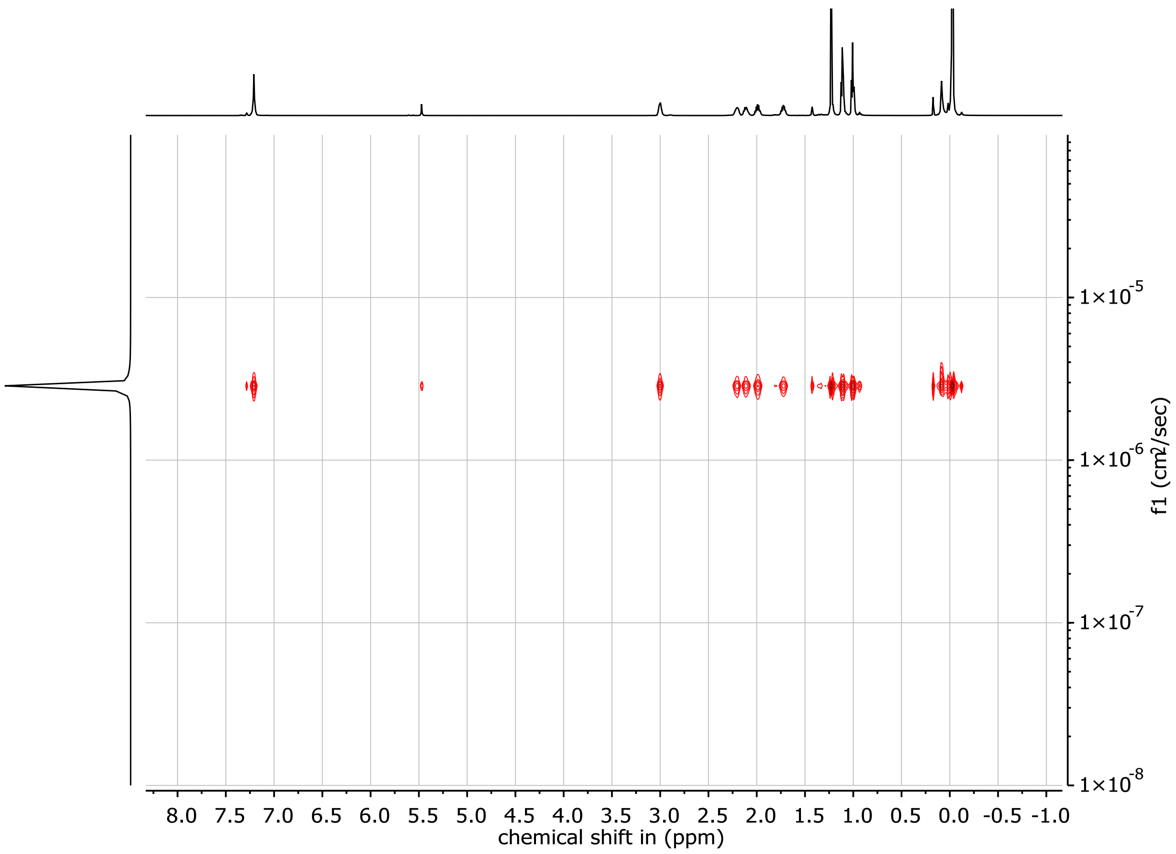

Figure S19. ^{1}H-DOSY NMR spectrum (600.15 MHz, 298 K, C_6D_{12}) of (BDI*)MgNa$_5$N''$_3$(P) (**1**).

Table S1. Results of the DOSY-experiment for complex **1** in C_6D_{12}

**DOSY analysis report**					
**entry**	**compound**	**log D**	**MW (est.)**	**MW (calc.)**	**comment**
**1**	$C_{61}H_{123}MgN_5Na_5PSi_6$ **1**	-9,5557	**1024**	1265,43	Δ ca. 20%
**2**	impurity 0.118 to 0.037 ppm	-9,4802	**774**		
**3**	residual H Signal C6D12	-8,9519			reference

All ^{1}H NMR signals belong to one species. MW estimation was executed following the Stalke-method described in literature.[4] Residual proton signal from deuterated solvent C_6D_{12} was taken as an internal reference with a log $D_{ref,fix}$-value of –8.8827. The signal was not influenced by overlapping. The DOSY unambiguously shows all signals of the complex within one horizontal line not indicating any equilibria due to dissociation/association.

The MW determination shows a 20% underestimation of the calculated MW which originates from limitations of Stalke´s method rather than from an accuracy problem. MW estimation with Stalke´s ECC method is valid for “small molecules”[5] and in case of the cyclohexane solvent calibration curves have been made with organic molecules in a molecular weight range from ca. 70 to 600 (see Supporting Information of ref. [4]). Furthermore, for MW determination in the present case the so-called “merge” calibration curve was applied, as the shape of the metal complex could not be classified exactly (e.g. ED, CS, DSE). “The merged calibration curves ... determines MW in a range of ΔMW = ± 23%.” (see ref. [5], page 3358).

Molecular weight determination:[6]

ECC concept: merge-C6D12

internal reference: C6D11H

6. Crystal structure determination

Suitable single crystals of compound **1** were embedded in protective perfluoropolyalkylether oil (viscosity 1800 cSt; ABCR GmbH) on a microscope slide and a single specimen was selected and subsequently transferred to the cold nitrogen gas stream of the diffractometer.

The intensity data was collected at 100 K using CuK_α radiation (λ = 1.54184 Å) on an Agilent SuperNova dual radiation diffractometer with microfocus X-ray sources and mirror optics. The measured data were processed with the CrysAlisPro software package.[7] Data were corrected for Lorentz and polarization effects, and an empirical absorption correction using spherical harmonics as well as a numerical absorption correction based on gaussian integration over a multifaceted crystal model were applied. Using Olex2,[8] the structure was solved by dual-space methods (SHELXT)[9] and refined by full-matrix least-squares procedures on F^2 using SHELXL.[10] All non-hydrogen atoms were refined with anisotropic displacement parameters. All hydrogen atoms were placed in geometrically calculated positions and refined by using a riding model where each H-atom was assigned a fixed isotropic displacement parameter with a value equal to 1.2U_{eq} (CH or CH_2) or 1.5U_{eq} (CH_3) of its parent C-atom.

Three out of four 3-pentyl groups of compound **1** and one of the co-crystallized *n*-pentane molecules were disordered. The disorder was modeled with the help of similarity restraints (SADI, SIMU) and rigid bond restraints (RIGU).[11] Additional DFIX restraints (1.52 Å) were used for C-C bonds of the disordered *n*-pentane moiety. The relative occupancies of the two alternative orientations of these groups were refined to 0.68(2)/0.32(2), (3-pentyl 1), 0.655(6)/0.345(6) (3-pentyl 2), 0.604(7)/0.396(7) (3-pentyl 3) and 0.718(7)/0.282(7) (*n*-pentane), respectively.

The crystal structure data has been deposited with the Cambridge Crystallographic Data Centre. CCDC 2467297 contains the supplementary crystallographic data for the complexes. This data can be obtained free of charge from The Cambridge Crystallographic Data Centre via www.ccdc.cam.ac.uk/data_request/cif.

Crystallographic and refinement data are summarized in **Table S2**.

Table S2: Crystal data and structure refinement for compound **1**.

Compound	**1**·2(*n*-pentane)
Identification code	hasj250327a
Empirical formula	$C_{71}H_{147}MgN_5Na_5PSi_6$
Formula weight	1409.70
Temperature/K	100.00(10)
Crystal system	monoclinic
Space group	$P2_1/n$
a/Å	12.0428(2)
b/Å	31.3027(4)
c/Å	24.1408(3)
α/°	90
β/°	99.3860(10)
γ/°	90
Volume/Å^3	8978.6(2)
Z	4
ρ_{calc}g/cm^3	1.043
μ/mm^{-1}	1.620
F(000)	3096.0
Crystal size/mm^3	0.43 × 0.34 × 0.11
Radiation	Cu Kα (λ = 1.54184)
2Θ range for data collection/°	7.754 to 143.714
Index ranges	-11 ≤ h ≤ 14, -26 ≤ k ≤ 38, -29 ≤ l ≤ 29
Reflections collected	67801
Independent reflections	17365 [R_{int} = 0.0328, R_{sigma} = 0.0274]
Data/restraints/parameters	17365/492/1011
Goodness-of-fit on F^2	1.061
Final R indexes [I>=2σ (I)]	R_1 = 0.0534, wR_2 = 0.1347
Final R indexes [all data]	R_1 = 0.0604, wR_2 = 0.1407
Largest diff. peak/hole / e Å^{-3}	0.85/-0.47

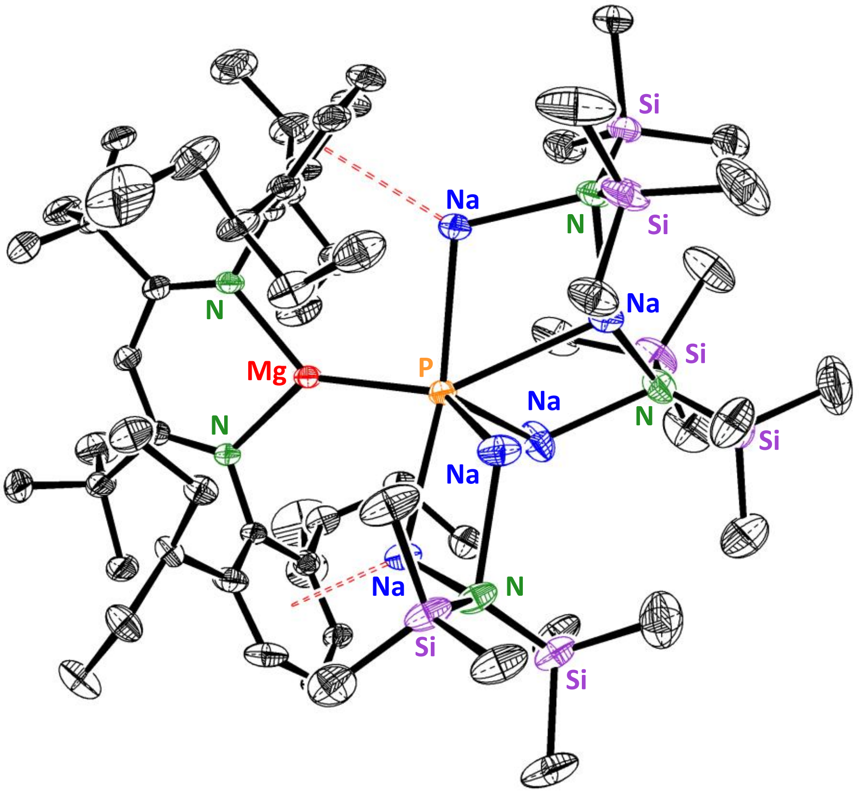

Figure S20. ORTEP representation of (BDI*)MgNa$_5$N''$_3$(P) (**1**). (CCDC 2467297) with atomic displacement ellipsoids set at 50% probability. Hydrogen atoms are omitted for clarity.

8. DFT calculations

All calculations were carried out using Gaussian 16A[12]. All methods were used as implemented. All structures were fully optimized at a B3PW91-GD3BJ/def2svp level of theory which includes Grimme D3 dispersion correction using Becke–Johnson dampening (GD3BJ)[13–17]. All structures were characterized as true minima (Nimag = 0) or as transition states (Nimag = 1) by frequency calculations on the same level of theory. Energies were determined at a B3PW91-GD3BJ/def2tzvp level of theory. The same level of theory was for the NPA charge calculations with NBO6[18]. QTAIM analysis was carried out using AIMAll (v17) with the wave functions obtained from the B3PW91-GD3BJ/def2tzvp level of theory[19,20].

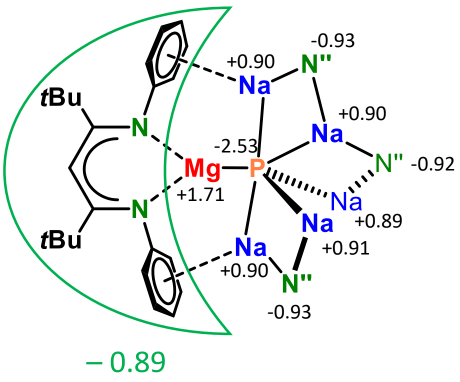

Figure S21. NPA charges for (BDI*)MgNa$_5$N''$_3$(P) (**1**).

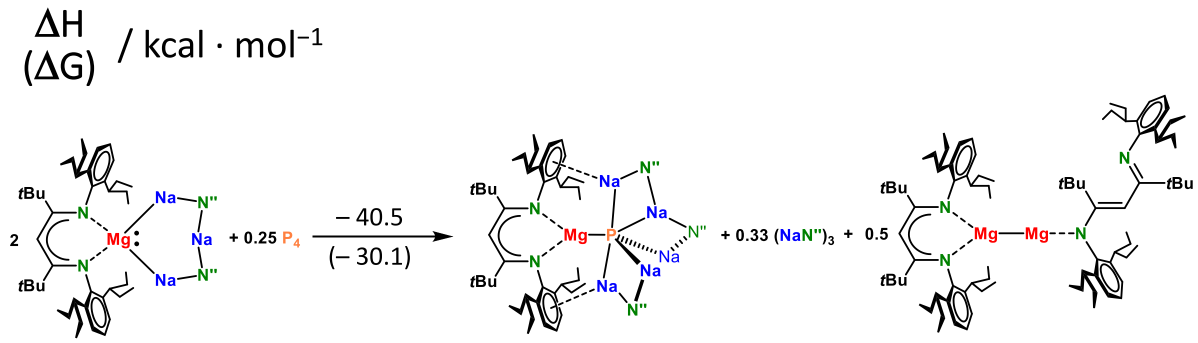

Figure S22. Reaction enthalpie (ΔH) and between brackets free energie (ΔG at 298 K) in kcal·mol^{-1}.

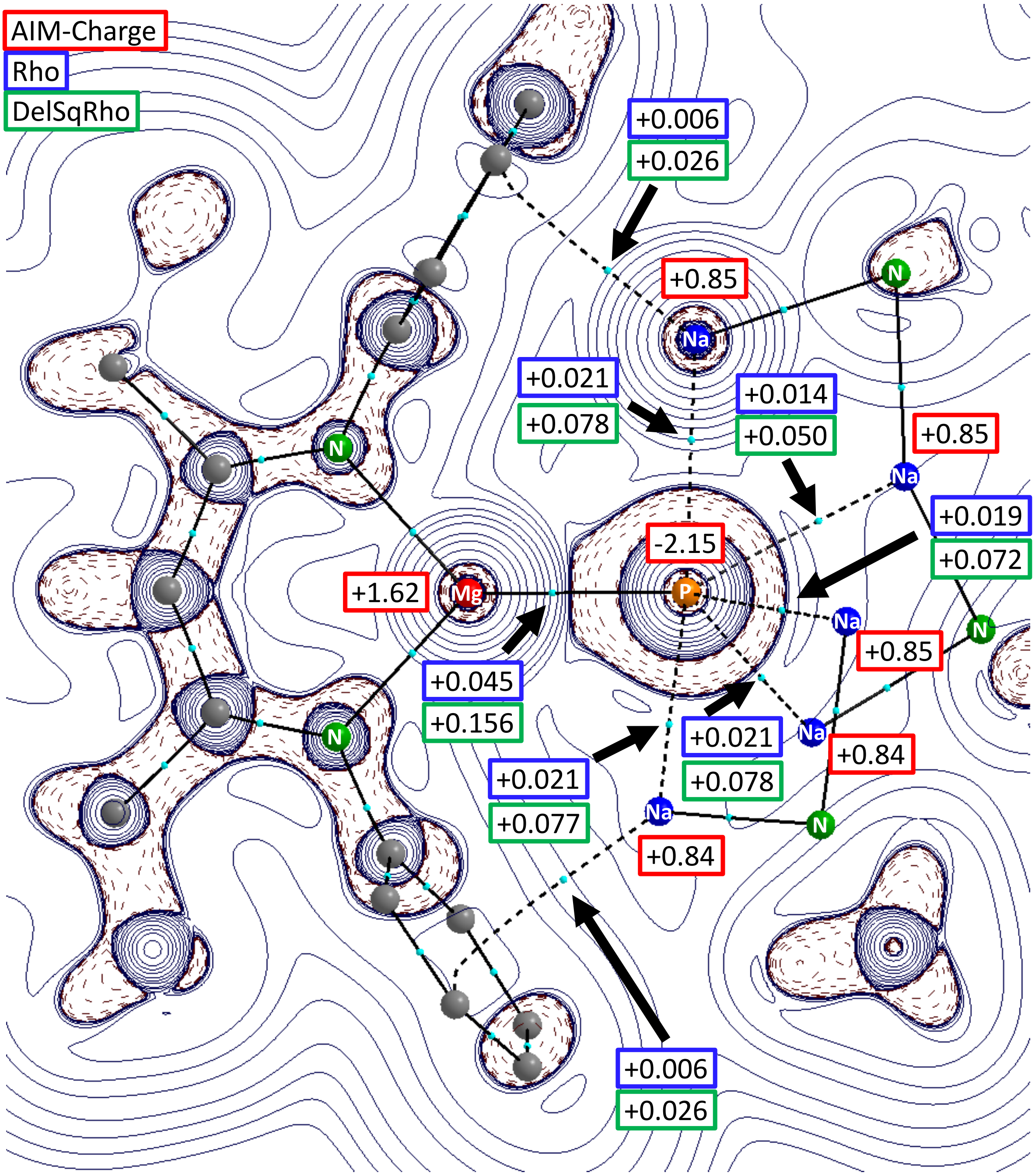

Figure S23: Laplacian distribution for (BDI*)MgNa$_5$N''$_3$(P) (**1**) showing bcps (light blue) with AIM charges (red boxes), $\rho(\mathbf{r})$ (e B^{-3} in blue boxes) and the Laplacian $\nabla^2\rho(\mathbf{r})$ (e B^{-5} in green boxes).

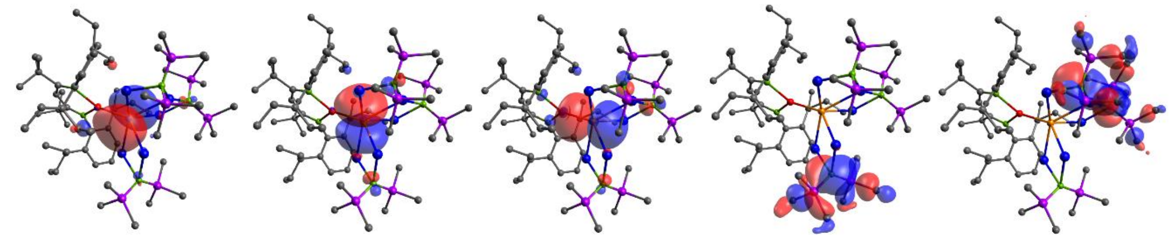

HOMO: −4.40 HOMO−1: −4.62 HOMO−2: −5.27 HOMO−3: −5.76 HOMO−4: −5.63

Atomic contributions to alpha molecular orbitals:

HOMO	HOMO−1	HOMO−2
P-p: 77.18 %,	P-p: 77.15 %,	P-p: 67.69 %,
Mg-p: 4.27 %,	Mg-p: 4.74 %,	Mg-s: 7.89 %,
Mg-d: 2.82 %,	Mg-d: 2.70 %,	Mg-p: 4.35 %
Na-s: 3.14 %,	Na-s: 1.85 %,	
Na-p: 2.06 %	Na-p: 1.30 %	

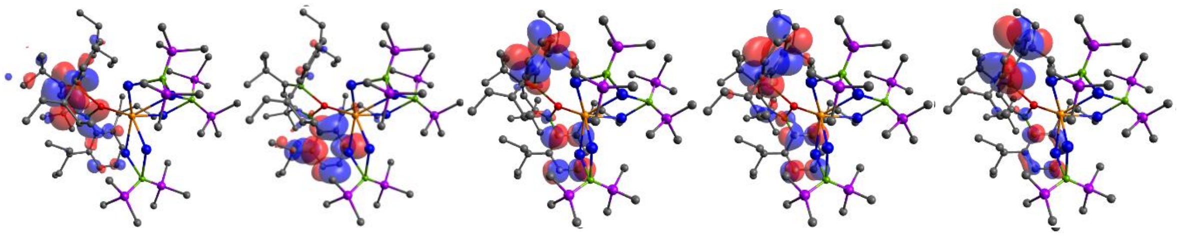

LUMO: −1.60 LUMO+1: −1.03 LUMO+2: −0.93 LUMO+3: −0.83 LUMO+4: −0.80

Figure S24: Selected MO's for (BDI*)MgNa$_5$N''$_3$(P) (**1**), computed at the B3PW91-D3BJ/def2tzvp//B3PW91-D3BJ/def2svp level of theory.

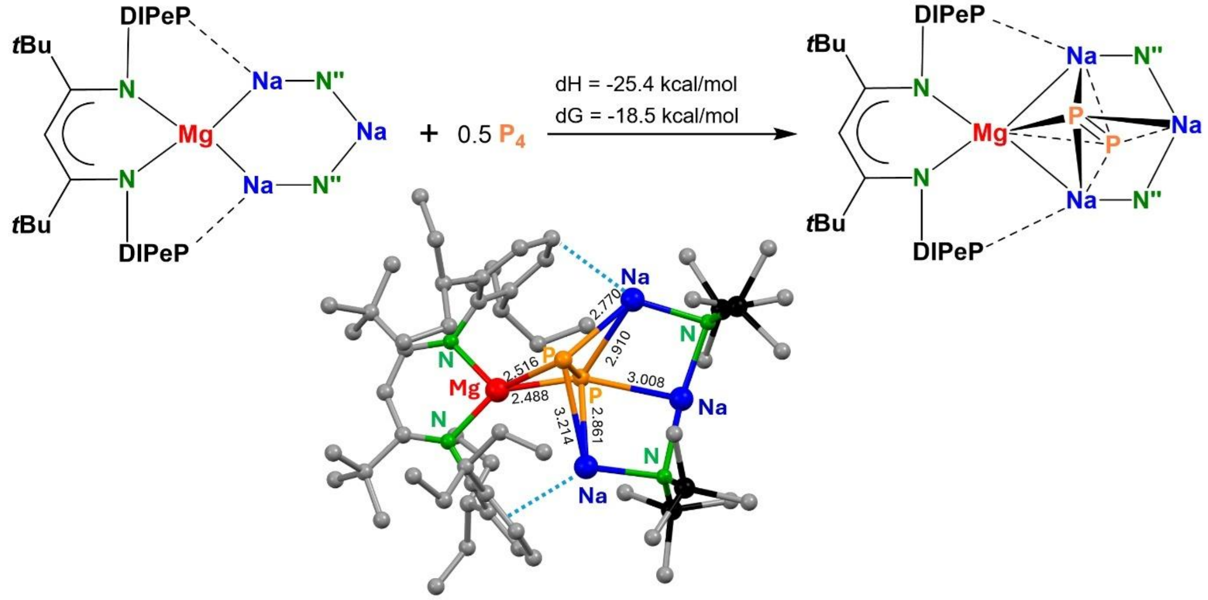

Figure S25: Reaction of (BDI*)MgNa$_5$N''$_3$(P) (**1**) with 0.5 equivalents of P_4 to give an encapculated P_2^{2-} anion. Note that the metallcycle is nearly too small to encapsulate this anion. One of the Na atoms show (η^6)Ar-Na bonding whereas the other shows only (η^1)Ar-Na bonding

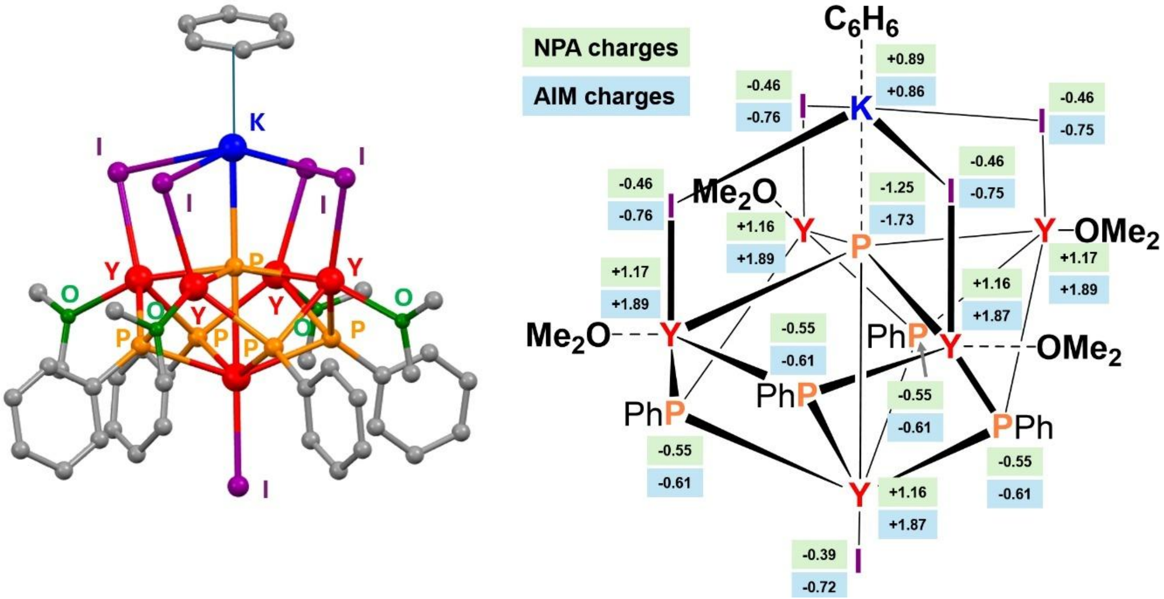

Figure S26: Optimized model system for the large Y phosphide cluster [K^+·(toluene)][$Y_5I_5(PAr)_4P^-$·$(THF)_4$] (**IV**) and the NPA and AIM charges.

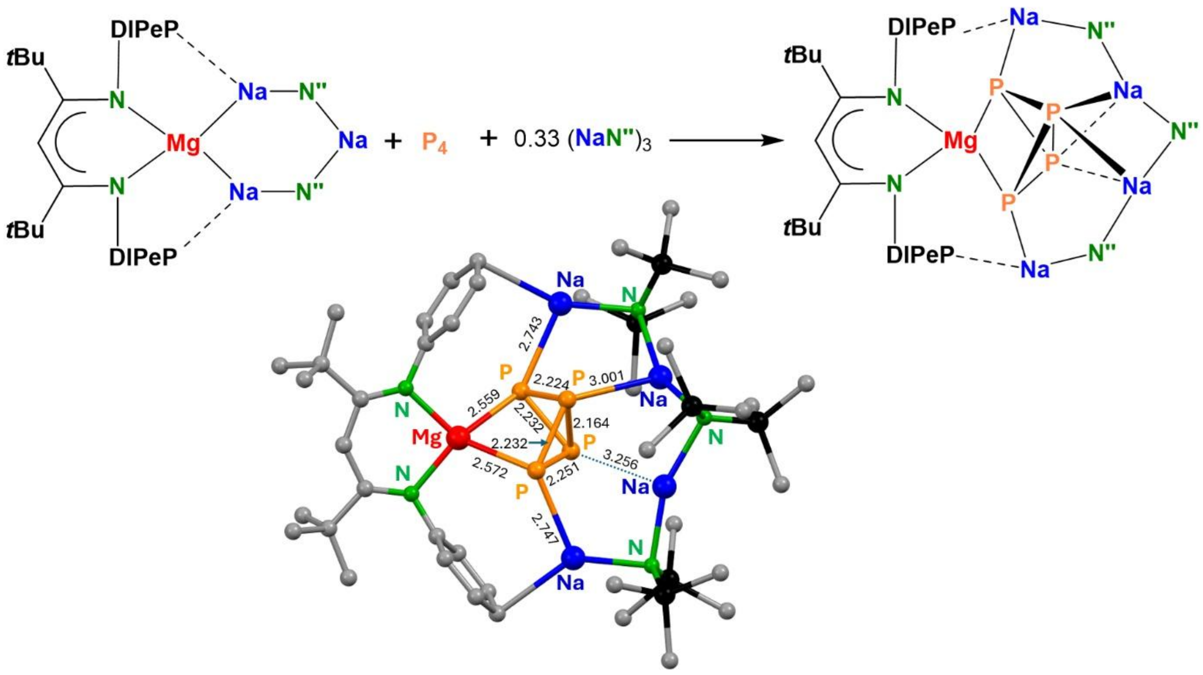

Figure S27: First step in the reaction of **VI** with P_4, resulting in encapsulation of P_4^{2-}. The original $MgNa_3$ metal cycle is too small and needed to be enlarged by one NaN'' unit. This reaction is exothermic: (ΔH = -81.6 kcal mol^{-1}, ΔG_{298K} = -62.7 kcal·mol^{-1}.

NBO & NLMO analysis for the Mg-P and Na-P contacts:

Using the Natural-Localized-Molecular-Orbital (NLMO) method (**Table S3**) all Mg-P and Na-P bonds are below the threshold and have been interpreted as a donor-acceptor bond between a lonepair at P^{3-} and the Na^{+} and Mg^{2+} cations with very little electron density on Na and Mg. Orbital occupancies (**Table S4**) clearly show that all valence orbitals of phosphorous are filled, while Na and Mg valence shells are predominantly empty.

Table S3: NLMOs of (BDI*)MgNa$_5$N''$_3$(P) (**1**) including the P valence electrons. Contributions of non-metals are neglectable.

**(2.00000) *e*⁻**<br>**96.7993% lonepair P**	**96.807% P** s (3.82%), **p 25.15 (96.15%),** d 0.01 (0.03%)<br>0.160% Mg s (0.42%), p99.99 (69.09%), d72.23 (30.49%)<br>0.450% Na1 s (92.94%), p 0.05 (4.34%), d 0.03 (2.71%)<br>**0.772% Na2 s (93.27%)**, p 0.04 (3.97%), d 0.03 (2.76%)<br>0.458% Na3 s (94.78%), p 0.03 (2.48%), d 0.03(2.74%)<br>0.021% Na4 s (11.82%), p 2.52 (29.83%), d 4.94 (58.35%)<br>0.067% Na5 s (69.95%), p 0.16 (11.34%), d 0.27 (18.71%)
**(2.00000) *e*⁻**<br>**95.2110% lonepair P**	**95.213% P** s (2.72%), **p35.81 (97.26%)**, d 0.01 (0.02%)<br>0.346% Mg s (5.17%), p13.66 (70.67%), d 4.67 (24.16%)<br>0.288% Na1 s (92.31%), p 0.03 (2.99%), d 0.05 (4.71%)<br>0.110% Na2 s (74.31%), p 0.08 (6.11%), d 0.26 (19.59%)<br>0.020% Na3 s (17.68%), p 1.86 (32.95%), d 2.79 (49.36%)<br>**1.106% Na4 s (96.94%)**, p 0.02 (1.82%), d 0.01 (1.23%)<br>0.411% Na5 s (93.34%), p 0.05 (4.22%), d 0.03 (2.44%)
**(2.00000) *e*⁻**<br>**94.5765% lonepair P**	**94.595% P s (31.31%), p 2.19 (68.66%),** d 0.00 (0.03%)<br>0.574% Mg s (78.86%), p 0.22 (17.38%), d 0.05 (3.76%)<br>0.386% Na1 s (96.23%), p 0.02 (1.90%), d 0.02 (1.87%)<br>0.189% Na2 s (87.48%), p 0.10 (8.38%), d 0.05(4.14%)<br>**0.905% Na3 s (96.60%)**, p 0.02 (1.58%), d 0.02 (1.82%)<br>**0.802% Na4 s (96.96%)**, p 0.01 (1.14%), d 0.02 (1.90%)<br>**1.782% Na5 s (98.44%)**, p 0.01 (0.78%), d 0.01 (0.78%)
**(2.00000) *e*⁻**<br>**88.3054% lonepair P**	**88.338% P s (61.73%), p 0.62 (38.18%)**, d 0.00 (0.09%)<br>**8.132% Mg s (95.35%)**, p 0.03 (3.21%), d 0.02 (1.45%)<br>**1.006% Na1 s (97.71%)**, p 0.02 (1.50%), d 0.01 (0.79%)<br>0.695% Na2 s (95.38%), p 0.02 (2.02%), d 0.03 (2.59%)<br>0.317% Na3 s (91.65%), p 0.06 (5.41%), d 0.03 (2.94%)<br>0.292% Na4 s (93.06%), p 0.03 (2.75%), d 0.05 (4.19%)<br>0.580% Na5 s (94.90%), p 0.02 (1.72%), d 0.04 (3.38%)

Table S4: Natural Population Analysis: Natural atomic valence orbital occupancies for (BDI*)$MgNa_5N''_3$(P) (**1**).

(BDI*)$MgNa_5N''_3$(P) (**1**)		
**atom**	**valence orbital**	**occupancy [e^-]**
P	3s<br>3p(x/y/z)	1.82516<br>1.86325/1.92643/1.90331
Mg	3s	0.25833
Na(1/2/3/4/5)	3s	0.09369/0.08756/0.08588/0.08774/0.10593

9. XYZ coordinates

```
202
Complex 1
P 0.676650 0.285104 -0.170452
Si 4.281247 -3.574761 -0.254011
Si 0.171471 4.089209 -3.583484
Mg -1.519518 -0.425993 0.313476
Si 1.740453 5.070559 -1.166889
Si 4.166332 -2.007926 -2.857803
Si 5.339113 1.676122 1.424807
Si 4.232819 -0.146479 3.611165
Na -0.374305 2.771113 -0.547146
Na 1.373088 -2.159158 -1.124396
Na 3.439147 -0.596146 0.272908
Na 1.848498 1.716051 -2.206515
Na 1.998620 1.438745 1.896491
N -3.192496 0.557205 1.113225
N -2.525693 -2.206105 -0.122620
N 3.661038 -2.337948 -1.263347
N 0.962471 3.859968 -2.086624
C -2.962458 1.949734 1.219626
C -2.205547 2.476435 2.298057
N 4.147402 0.654598 2.100691
C -1.843363 3.830994 2.269066
H -1.292929 4.256394 3.108900
C -3.387834 2.803939 0.161989
C -4.345832 -0.082454 1.304219
C -3.799861 -2.390117 0.205504
C -1.646992 -3.184480 -0.655296
C -1.385926 -3.246490 -2.046802
C -1.886443 1.629710 3.514286
H -2.394916 0.666062 3.358705
C -2.197428 4.658222 1.205826
H -1.892049 5.706516 1.196523
C -0.394825 1.286801 3.656162
H -0.294704 0.532310 4.449477
H -0.093369 0.775067 2.722591
C -4.563115 -1.409415 0.882597
H -5.557730 -1.758056 1.122022
C -2.979016 4.144359 0.169893
H -3.285633 4.805581 -0.641336
C -0.442836 -4.177550 -2.512454
H -0.256951 -4.262191 -3.584019
C -0.953866 -4.037719 0.245218
C 0.545869 2.443709 3.975484
H 0.635055 3.164273 3.147373
H 1.555832 2.080614 4.228303
H 0.212635 3.015963 4.853414
C -3.909117 -4.838675 -0.783926
H -3.453047 -4.581797 -1.744638
H -4.647019 -5.633616 -0.972464
H -3.129219 -5.260149 -0.142040
C -4.645827 -3.659243 -0.138596
C -2.474270 2.243449 4.797147
H -2.002755 3.218498 5.003690
H -3.537102 2.470871 4.620682
C 0.253678 -5.006731 -1.634920
H 0.990455 -5.715541 -2.017445
C -5.501301 1.989487 2.445218
H -6.388366 2.234644 3.049174
H -5.522573 2.627968 1.556822
H -4.621150 2.262206 3.032548
C -5.553738 0.496090 2.109032
C -1.274193 -3.977593 1.725210
H -2.355889 -3.779494 1.796335
C 4.284856 -5.302440 -1.029716
H 3.284806 -5.562217 -1.410659
H 4.588585 -6.074007 -0.303229
H 4.986473 -5.347953 -1.878268
C -4.292373 2.265805 -0.931169
H -4.976404 1.553356 -0.443030
C 3.172659 -3.672678 1.289508
H 3.133500 -2.726772 1.852959
H 3.528429 -4.442234 1.993205
H 2.137485 -3.932101 1.015690
C -3.528774 1.455723 -1.994316
H -3.097114 2.148121 -2.736405
H -2.661406 0.946070 -1.539806
C -0.004534 -4.929978 -0.265690
H 0.532860 -5.592031 0.414237
C 1.111601 5.164222 -4.823132
H 1.233864 6.191005 -4.441943
H 0.590719 5.226464 -5.792662
H 2.119494 4.755818 -5.002126
C 6.045629 -3.225640 0.334964
H 6.750468 -3.280465 -0.509253
H 6.379875 -3.941069 1.103718
H 6.124398 -2.214004 0.763517
C -5.304323 -4.209668 1.142294
H -4.548411 -4.426245 1.911975
H -5.827328 -5.149974 0.910766
H -6.042463 -3.526107 1.581698
C -2.133021 -2.363893 -3.024659
H -2.990299 -1.950391 -2.474098
C -1.566134 4.828578 -3.394541
H -2.183484 4.198599 -2.734638
H -2.091013 4.917959 -4.359747
H -1.513077 5.830088 -2.939270
C -0.064545 2.378130 -4.384745
```

H	0.896184	1.948556	-4.721405
H	-0.712535	2.423291	-5.274434
H	-0.528742	1.669732	-3.677412
C	-5.732244	-3.218117	-1.139843
H	-6.375642	-2.423679	-0.738629
H	-6.368874	-4.077377	-1.403103
H	-5.273726	-2.843709	-2.067646
C	3.421217	-0.337434	-3.407930
H	2.334384	-0.357151	-3.227708
H	3.588466	-0.136996	-4.477791
H	3.891213	0.476989	-2.831511
C	-1.006524	-5.278723	2.488357
H	0.068004	-5.523511	2.430647
H	-1.193704	-5.078254	3.556472
C	-4.421285	0.426764	-2.668500
H	-5.335038	0.886308	-3.074859
H	-4.728735	-0.343819	-1.945116
H	-3.911839	-0.068920	-3.502478
C	0.776409	6.699147	-1.067249
H	0.677199	7.161550	-2.062640
H	1.269538	7.430604	-0.406270
H	-0.241396	6.521847	-0.683207
C	-0.566166	-2.798391	2.424540
H	-0.259692	-2.023660	1.695222
H	0.391415	-3.152558	2.839384
C	1.915105	4.451367	0.630748
H	0.941449	4.243937	1.107753
H	2.431012	5.191154	1.262389
H	2.524964	3.534387	0.636664
C	-5.574732	-0.268019	3.448823
H	-4.637354	-0.106184	4.002389
H	-5.696116	-1.350312	3.304596
H	-6.404390	0.093800	4.076548
C	2.511720	-0.869153	4.011512
H	1.884648	-0.146797	4.557463
H	2.592308	-1.763325	4.649934
H	1.964064	-1.164271	3.100649
C	-1.845187	-6.471305	2.052255
H	-2.920509	-6.265377	2.169253
H	-1.611733	-7.362828	2.653899
H	-1.672906	-6.730425	0.996481
C	-6.879211	0.267138	1.357622
H	-7.154827	-0.791907	1.269558
H	-6.838509	0.688892	0.341876
H	-7.696784	0.768894	1.897004
C	4.933991	1.877129	-0.431783
H	5.112018	0.939996	-0.986743
H	5.553547	2.652053	-0.908497
H	3.878598	2.172714	-0.564101
C	4.677924	1.007837	5.049900
H	5.710072	1.380484	4.944754
H	4.605436	0.496790	6.024309
H	4.013474	1.887526	5.075310
C	5.460558	-1.582670	3.684767
H	5.198036	-2.377359	2.971738
H	5.481963	-2.027773	4.693477
H	6.479588	-1.244117	3.442193
C	3.492049	5.465518	-1.754268
H	4.109603	4.553447	-1.771917
H	3.994269	6.201022	-1.104920
H	3.469741	5.872496	-2.777704
C	-2.351284	1.345282	6.020331
H	-2.887079	1.774323	6.880423
H	-1.304466	1.200784	6.327545
H	-2.777098	0.347528	5.825632
C	6.036267	-1.852787	-3.094276
H	6.462071	-1.129837	-2.379619
H	6.293314	-1.521107	-4.113680
H	6.534391	-2.820346	-2.921762
C	3.544974	-3.288688	-4.112362
H	4.008602	-4.268709	-3.918754
H	3.774055	-3.003094	-5.152203
H	2.454140	-3.425518	-4.031401
C	-5.163758	3.334144	-1.603739
H	-4.517052	4.084993	-2.088182
H	-5.702999	2.851215	-2.433860
C	7.105999	1.007741	1.507454
H	7.447259	0.929168	2.552354
H	7.813031	1.663132	0.972816
H	7.166848	0.002318	1.061080
C	5.366337	3.414399	2.175394
H	4.369856	3.881775	2.146672
H	6.066744	4.081630	1.646850
H	5.677027	3.363506	3.231073
C	-1.285211	-1.151106	-3.462784
H	-1.939735	-0.437332	-3.986550
H	-0.929888	-0.622865	-2.557603
C	-2.694866	-3.177097	-4.202756
H	-1.869189	-3.575746	-4.813874
H	-3.211016	-4.063479	-3.797799
C	-1.421706	-2.178423	3.517723
H	-0.866472	-1.412889	4.074712
H	-1.759443	-2.934475	4.243765
H	-2.325997	-1.711469	3.092315
C	-6.176287	4.015461	-0.694340
H	-5.693084	4.527048	0.151901
H	-6.888895	3.287109	-0.277037
H	-6.759372	4.768022	-1.246496
C	-0.088917	-1.451675	-4.350858
H	0.413206	-0.518862	-4.642050
H	-0.372650	-1.969673	-5.279964
H	0.663145	-2.078987	-3.850110

C	-3.652445	-2.408077	-5.101130
H	-4.527985	-2.047057	-4.539390
H	-4.024129	-3.046735	-5.916803
H	-3.171170	-1.533347	-5.564742

202

Intermediate exchange long/short arms

P	-0.590411	0.065627	-0.041919
Si	-2.279222	4.970572	1.076523
Si	-2.535420	-3.110536	-3.281018
Mg	1.747395	-0.017422	0.220266
Si	-3.299251	-4.351218	-0.631518
Si	-3.205309	3.956642	-1.660289
Si	-5.849534	-0.376476	0.248557
Si	-4.482565	0.214165	2.780122
Na	-0.479893	-2.491614	-0.985905
Na	-0.392756	2.734711	-0.482221
Na	-2.913540	1.703661	0.618122
Na	-3.034284	-0.783086	-1.127585
Na	-2.227289	-1.280808	1.645980
N	3.020135	-1.549358	0.846669
N	3.303550	1.253212	-0.359552
N	-2.400063	3.804075	-0.163568
N	-2.660785	-3.076829	-1.573381
C	2.268990	-2.733104	1.027779
C	1.483131	-2.911680	2.193265
N	-4.356929	-0.116116	1.089049
C	0.626463	-4.017921	2.259680
H	0.053964	-4.199606	3.170183
C	2.199319	-3.672161	-0.038503
C	4.343947	-1.419884	0.874037
C	4.585079	0.932072	-0.221921
C	2.786504	2.514208	-0.747421
C	2.318784	2.730860	-2.067545
C	1.639983	-1.982357	3.380957
H	2.471217	-1.308038	3.127037
C	0.507015	-4.912139	1.199811
H	-0.183114	-5.754914	1.265502
C	0.419461	-1.071197	3.612283
H	0.711673	-0.263078	4.301259
H	0.179494	-0.571088	2.653394
C	5.005413	-0.280810	0.371920
H	6.080844	-0.351472	0.458372
C	1.296509	-4.739149	0.062591
H	1.222364	-5.461889	-0.752392
C	1.720150	3.964153	-2.371022
H	1.384570	4.159398	-3.390267
C	2.640859	3.521647	0.245353
C	-0.827463	-1.741542	4.170694
H	-1.216077	-2.534336	3.511183
H	-1.627789	-1.005323	4.329868
H	-0.649529	-2.221825	5.144519
C	5.457963	3.179048	-1.295982
H	4.783210	3.139613	-2.156377
H	6.399504	3.636424	-1.637073
H	5.017276	3.850456	-0.551993
C	5.785970	1.791079	-0.734193
C	2.060809	-2.754777	4.642649
H	1.258535	-3.446412	4.949040
H	2.916197	-3.401056	4.388767
C	1.551985	4.950249	-1.400192
H	1.065723	5.893810	-1.655045
C	4.711489	-3.778676	1.975562
H	5.494413	-4.360308	2.486100
H	4.361840	-4.372988	1.125461
H	3.876719	-3.669850	2.673489
C	5.314255	-2.433760	1.558048
C	3.198019	3.280454	1.635004
H	4.143487	2.733640	1.490006
C	-1.819868	6.704364	0.464707
H	-0.877052	6.669654	-0.104836
H	-1.692977	7.418606	1.294872
H	-2.597586	7.104163	-0.206205
C	3.101518	-3.510139	-1.249896
H	4.065901	-3.134657	-0.873195
C	-0.901375	4.436393	2.272589
H	-1.042616	3.402854	2.627976
H	-0.847227	5.090193	3.158093
H	0.079910	4.480364	1.774051
C	2.584518	-2.441012	-2.229453
H	1.826950	-2.898875	-2.889497
H	2.053704	-1.642758	-1.681728
C	2.014088	4.724206	-0.102534
H	1.894834	5.507305	0.647192
C	-3.988298	-3.886188	-4.205303
H	-4.079902	-4.960503	-3.979030
H	-3.865263	-3.781987	-5.295731
H	-4.934128	-3.399685	-3.919393
C	-3.873315	5.137085	2.089211
H	-4.695236	5.495955	1.449650
H	-3.763726	5.840817	2.930684
H	-4.186606	4.164972	2.504389
C	6.803562	2.009231	0.402730
H	6.325449	2.474849	1.277627
H	7.600847	2.684539	0.056748
H	7.285661	1.082149	0.739427
C	2.507416	1.685132	-3.147276
H	3.224173	0.954136	-2.744285
C	-0.965904	-4.019432	-3.863067
H	-0.046165	-3.520928	-3.511004
H	-0.899785	-4.060030	-4.962653
H	-0.949181	-5.052907	-3.481938

C	-2.342443	-1.325205	-3.899661
H	-3.275285	-0.744864	-3.798195
H	-2.063165	-1.291415	-4.964426
H	-1.551519	-0.799655	-3.335669
C	6.444754	0.996269	-1.879570
H	6.786408	0.003447	-1.557224
H	7.313243	1.550786	-2.268482
H	5.736453	0.856428	-2.710182
C	-3.486113	2.193440	-2.330021
H	-2.547464	1.612714	-2.291201
H	-3.849676	2.179185	-3.369220
H	-4.247673	1.681175	-1.717414
C	3.540166	4.555679	2.412026
H	2.626168	5.158055	2.551476
H	3.837868	4.252945	3.429751
C	3.695580	-1.811980	-3.053214
H	4.289883	-2.571346	-3.583744
H	4.377674	-1.246987	-2.400096
H	3.295844	-1.124223	-3.808253
C	-2.406119	-6.001894	-0.917492
H	-2.643817	-6.386801	-1.923037
H	-2.702740	-6.773824	-0.188373
H	-1.313390	-5.879185	-0.865233
C	2.300011	2.362994	2.492332
H	1.614956	1.767882	1.858589
H	1.617030	2.987623	3.090069
C	-3.121239	-3.902920	1.210045
H	-2.070868	-3.690068	1.473273
H	-3.456277	-4.717701	1.870676
H	-3.767091	-3.033932	1.420993
C	5.808349	-1.743615	2.846075
H	4.962987	-1.520885	3.514714
H	6.327523	-0.798732	2.634919
H	6.501209	-2.407249	3.387265
C	-4.538488	-1.363764	3.839794
H	-5.482540	-1.896286	3.647671
H	-4.501354	-1.116461	4.913379
H	-3.731062	-2.087209	3.644232
C	4.648485	5.403126	1.803639
H	5.587601	4.833048	1.728470
H	4.850408	6.294273	2.417136
H	4.392609	5.749345	0.790750
C	6.513730	-2.752877	0.646150
H	7.166594	-1.888560	0.466698
H	6.180627	-3.130533	-0.332449
H	7.132429	-3.533569	1.114160
C	-5.610102	-1.022175	-1.527684
H	-5.199305	-0.262081	-2.214118
H	-6.610879	-1.261292	-1.919787
H	-5.011944	-1.943168	-1.610854
C	-5.930784	1.273281	3.368240

H	-5.942646	2.246122	2.852829
H	-5.831630	1.464996	4.449328
H	-6.904551	0.788788	3.202305
C	-2.945228	1.207156	3.344381
H	-2.845989	1.159979	4.440232
H	-3.077220	2.275844	3.104781
H	-1.973452	0.905041	2.913012
C	-5.142421	-4.715368	-0.858855
H	-5.761033	-3.834039	-0.634498
H	-5.460668	-5.528745	-0.185710
H	-5.366786	-5.026642	-1.890164
C	2.440855	-1.860558	5.814349
H	2.800631	-2.456469	6.666856
H	1.590309	-1.259566	6.170550
H	3.244483	-1.160052	5.535607
C	-4.898151	4.795668	-1.572833
H	-5.550316	4.286890	-0.845174
H	-5.409256	4.793423	-2.549522
H	-4.796201	5.844504	-1.249817
C	-2.188902	4.897144	-2.955351
H	-2.033566	5.936955	-2.625726
H	-2.684115	4.920105	-3.939922
H	-1.193850	4.444374	-3.089812
C	3.389227	-4.817700	-1.996450
H	2.442556	-5.242067	-2.373021
H	3.965391	-4.567516	-2.901430
C	-6.846101	1.215485	0.004305
H	-7.222672	1.623503	0.952792
H	-7.712093	1.041241	-0.655153
H	-6.222533	1.993354	-0.465373
C	-6.971989	-1.644144	1.097185
H	-6.437363	-2.592490	1.263738
H	-7.863232	-1.860493	0.486260
H	-7.322879	-1.282845	2.076623
C	1.209611	0.899206	-3.430507
H	1.454693	0.049911	-4.089352
H	0.847256	0.456880	-2.483368
C	3.140449	2.294385	-4.410303
H	2.430746	2.986920	-4.890940
H	3.996448	2.919425	-4.105680
C	3.118781	1.448670	3.389917
H	2.482631	0.869875	4.072400
H	3.824195	2.024821	4.009176
H	3.713101	0.742442	2.788538
C	4.157164	-5.862138	-1.198895
H	3.619368	-6.161563	-0.286608
H	5.142608	-5.479611	-0.890740
H	4.327653	-6.770349	-1.796612
C	0.065090	1.689716	-4.043285
H	-0.785345	1.028650	-4.257476
H	0.343732	2.184396	-4.987087

H -0.309733 2.468293 -3.363467
C 3.609528 1.270077 -5.433200
H 4.366477 0.593961 -5.005328
H 4.063896 1.763972 -6.305614
H 2.780602 0.648441 -5.804517

174
(BDI*)MgNa3N’’2(P2)
Si -3.347335 3.129503 -2.023721
Si -3.885935 3.344407 0.992535
Mg 1.377558 -0.194097 0.032269
Na -1.048436 2.250493 -0.002747
Na -3.872315 0.340055 -0.272064
N 2.868958 1.226924 -0.011180
N 2.649240 -1.785965 -0.187387
N -3.252045 2.582414 -0.404009
C 4.158234 0.910395 -0.121221
C 4.612381 -0.422266 -0.234273
H 5.689372 -0.499745 -0.321330
C 3.963062 -1.680569 -0.254291
C 5.320958 1.948187 -0.082228
C 4.901305 3.420224 -0.043517
H 4.338015 3.729230 -0.929912
H 5.812428 4.036425 -0.002208
H 4.303465 3.663546 0.839597
C 6.132279 1.679022 1.200380
H 5.497056 1.788100 2.092066
H 6.956750 2.404740 1.281254
H 6.562957 0.668978 1.218676
C 6.224595 1.773104 -1.317165
H 6.716957 0.792629 -1.353648
H 7.014219 2.540108 -1.307416
H 5.650823 1.892821 -2.248600
C 4.913990 -2.888960 -0.466634
C 4.221555 -4.250332 -0.399217
H 3.775495 -4.453975 0.580149
H 4.966717 -5.036403 -0.594834
H 3.435500 -4.347833 -1.153973
C 6.048353 -2.885817 0.571571
H 6.679587 -1.989128 0.511482
H 6.698529 -3.759313 0.409445
H 5.652318 -2.948586 1.596277
C 5.505187 -2.744638 -1.883220
H 4.704916 -2.730107 -2.638669
H 6.164087 -3.599358 -2.103316
H 6.090770 -1.822187 -1.997460
C 2.237757 2.490998 -0.073958
C 1.891913 3.013589 -1.353397
C 1.060233 4.137468 -1.419278
H 0.772213 4.537266 -2.392777
C 0.614027 4.777455 -0.260614
H -0.029477 5.656806 -0.329273
C 1.017640 4.300725 0.986415
H 0.714737 4.840212 1.884849
C 1.818130 3.152516 1.111053
C 2.500095 2.390591 -2.595807
H 3.518865 2.094150 -2.300400
C 1.804812 1.095295 -3.060956
H 1.057409 1.343107 -3.832937
H 1.201372 0.654563 -2.247980
C 2.807985 0.076879 -3.581306
H 3.450182 0.513746 -4.362817
H 2.312710 -0.800644 -4.015915
H 3.464649 -0.268160 -2.767560
C 2.650168 3.360444 -3.772804
H 1.654724 3.725822 -4.078669
H 3.014725 2.777264 -4.634700
C 2.301331 2.703393 2.477327
H 3.093943 1.960881 2.293412
C 2.941564 3.870374 3.251204
H 3.608979 4.424523 2.571113
H 2.166008 4.594039 3.550802
C 3.727767 3.434399 4.479337
H 4.207843 4.295743 4.968030
H 4.522630 2.720193 4.210252
H 3.086658 2.947799 5.229647
C 1.224874 1.967683 3.296546
H 0.907238 1.082344 2.723685
H 1.700570 1.571773 4.207825
C -0.015003 2.757936 3.682873
H 0.217344 3.663936 4.264690
H -0.686225 2.136901 4.294004
H -0.598847 3.076859 2.806357
C 1.775762 -2.848640 0.128917
C 0.911017 -3.373677 -0.862124
C -0.191090 -4.135517 -0.444413
H -0.841618 -4.588091 -1.193774
C -0.452608 -4.351859 0.906075
H -1.313521 -4.956652 1.209462
C 0.432920 -3.860323 1.868193
H 0.250243 -4.076737 2.922272
C 1.581595 -3.148812 1.504230
C 1.246731 -3.245104 -2.336546
H 2.219486 -2.729227 -2.380978
C 0.283827 -2.378698 -3.169678
H 0.254304 -1.374263 -2.721532
H 0.734685 -2.252128 -4.167551
C -1.142491 -2.877533 -3.333990
H -1.697030 -2.221556 -4.021022
H -1.687666 -2.861426 -2.381198
H -1.192047 -3.898524 -3.744491
C 1.446128 -4.645069 -2.950328

H	2.069953	-5.244473	-2.267855
H	0.479621	-5.173358	-2.992476
C	2.082451	-4.630654	-4.332910
H	3.043099	-4.089895	-4.322122
H	1.438244	-4.144217	-5.080728
H	2.280886	-5.653446	-4.687970
C	2.647667	-2.769964	2.524306
H	3.604463	-2.808711	1.980817
C	2.756983	-3.781399	3.672982
H	3.503318	-3.402412	4.388851
H	1.808232	-3.802485	4.236045
C	3.152278	-5.189138	3.249043
H	4.149173	-5.195176	2.780617
H	2.443105	-5.612417	2.521548
H	3.190894	-5.869939	4.113101
C	2.537967	-1.336327	3.066034
H	1.724359	-1.288572	3.809252
H	2.213090	-0.667598	2.258348
C	3.841102	-0.794763	3.633693
H	3.707057	0.224908	4.023393
H	4.610967	-0.750393	2.847476
H	4.233271	-1.408440	4.459360
C	-2.914885	4.898322	1.494432
H	-1.848634	4.673125	1.661422
H	-3.307284	5.337233	2.426406
H	-2.969064	5.665344	0.706290
C	-3.732156	2.125235	2.438760
H	-4.447675	1.293259	2.346801
H	-3.931179	2.608000	3.408798
H	-2.726113	1.674944	2.492384
C	-5.701725	3.849052	0.842127
H	-5.827872	4.620873	0.065434
H	-6.098567	4.253731	1.787445
H	-6.324059	2.985972	0.555119
Si	-5.125831	-2.475794	-1.126598
Si	-5.704624	-1.717683	1.800422
Na	-2.686200	-2.334681	1.041982
N	-4.777917	-1.707580	0.361967
C	3.594891	4.528781	-3.529498
H	3.274411	5.146483	-2.676700
H	3.652329	5.184847	-4.411299
H	4.615086	4.174010	-3.314539
C	-1.997796	2.244763	-3.029193
H	-0.985752	2.551627	-2.713304
H	-2.076343	2.470091	-4.105048
H	-2.059498	1.150173	-2.909283
C	-3.046187	4.989672	-2.233647
H	-3.826497	5.577912	-1.724097
H	-3.044862	5.283575	-3.296202
C	-5.010516	2.743746	-2.840752
H	-5.212851	1.660519	-2.846272
H	-5.047134	3.091137	-3.886157
H	-5.830051	3.231983	-2.289732
C	-4.227493	-4.149199	-1.245081
H	-3.136676	-4.027011	-1.133093
H	-4.393181	-4.653684	-2.210916
H	-4.578109	-4.824709	-0.446835
C	-4.487079	-1.402147	-2.556973
H	-5.080949	-0.476830	-2.650887
H	-4.561796	-1.925556	-3.523249
H	-3.428253	-1.128205	-2.416493
C	-6.952870	-2.804000	-1.490066
H	-7.372865	-3.566621	-0.816839
H	-7.545416	-1.882731	-1.368028
C	-6.928195	-0.275480	1.891018
H	-6.430545	0.700582	1.777389
H	-7.483746	-0.257136	2.842881
H	-7.661397	-0.366894	1.072905
H	-2.075520	5.278797	-1.799634
H	-7.089083	-3.158403	-2.525133
C	-6.705615	-3.297916	2.110124
H	-7.547197	-3.393145	1.407230
H	-7.121972	-3.305137	3.131202
H	-6.072631	-4.193960	1.998711
C	-4.509186	-1.596358	3.280177
H	-3.987118	-2.557773	3.444857
H	-5.045907	-1.376183	4.216990
H	-3.743183	-0.813930	3.149877
P	-1.014870	-0.558716	-0.545772
P	-0.622267	-0.548796	1.517397

108

Yttrium cluster

O	0.462267	4.764720	0.995948
Y	-0.439491	2.670866	0.396130
Y	2.262459	0.045257	0.194577
P	1.045751	1.567555	2.296789
P	-0.641207	-0.050777	-0.138544
C	1.874305	4.981279	0.947633
C	-0.242161	5.784892	1.690137
C	1.902719	2.707286	3.426901
C	3.305334	2.812106	3.419528
C	3.952315	3.751445	4.222240
H	5.043005	3.820275	4.194967
C	3.217253	4.602530	5.050950
H	3.726695	5.340132	5.675626
C	1.824102	4.495915	5.079856
H	1.238600	5.148874	5.733118
C	1.173379	3.556769	4.280003
I	5.136584	0.142408	0.517821
I	-3.303850	3.196319	0.813093

K	-3.778149	-0.165489	-0.525794
O	0.977641	-4.787772	-0.960308
Y	-0.150873	-2.756682	-0.566937
P	1.637379	-1.548132	-2.112086
C	2.346731	-4.933078	-0.577790
C	0.523825	-5.809740	-1.837381
C	2.809781	-2.613883	-3.008519
C	4.173103	-2.638742	-2.662299
C	5.051661	-3.518244	-3.294744
H	6.105645	-3.524764	-3.004640
C	4.592655	-4.388087	-4.286765
H	5.282560	-5.078538	-4.777781
C	3.243983	-4.360649	-4.651634
H	2.875114	-5.028606	-5.434978
C	2.362634	-3.481234	-4.022986
I	-2.841622	-3.511923	-1.493073
O	1.266669	1.057172	-4.705229
Y	0.016530	0.454880	-2.799610
P	1.436992	2.240909	-1.457077
C	2.667556	0.788485	-4.774884
C	0.867360	2.171347	-5.495258
C	2.542729	3.424547	-2.288091
C	3.936971	3.234754	-2.287546
C	4.778316	4.133321	-2.943605
H	5.858443	3.965246	-2.929336
C	4.250450	5.240384	-3.611996
H	4.911940	5.941841	-4.125962
C	2.868563	5.446095	-3.611137
H	2.443727	6.312828	-4.125004
C	2.024066	4.550744	-2.954714
I	-2.700690	1.086567	-3.734944
O	0.214544	-1.036981	4.769078
Y	-0.579068	-0.521110	2.609670
P	1.230965	-2.204093	1.644328
C	1.573227	-0.762319	5.114781
C	-0.417972	-1.955859	5.650179
C	2.165871	-3.247045	2.806480
C	3.534682	-3.021399	3.040118
C	4.230207	-3.780026	3.981498
H	5.292394	-3.583736	4.149858
C	3.579079	-4.780296	4.707367
H	4.125909	-5.370753	5.446380
C	2.223135	-5.023795	4.473837
H	1.704758	-5.810738	5.028705
C	1.524534	-4.268625	3.531873
I	-3.404807	-1.254425	2.945309
C	-7.090838	0.958954	-1.134558
C	-6.852867	1.448428	0.154775
H	-6.729342	2.521218	0.318302
C	-6.747588	0.561429	1.231458
H	-6.534732	0.940702	2.233460
H	-6.786533	-1.506754	1.859688
C	-6.885358	-0.814883	1.020269
C	-7.123849	-1.304980	-0.268533
H	-7.219979	-2.380756	-0.434683
C	-7.225364	-0.417856	-1.346561
H	-7.404069	-0.800746	-2.354519
H	-7.161253	1.651417	-1.976994
H	1.307199	-3.464657	-4.309796
H	4.541297	-1.971686	-1.877142
H	-0.526874	-5.600251	-2.080026
H	1.129153	-5.808889	-2.757802
H	0.596376	-6.791670	-1.341647
H	2.984462	-4.957695	-1.474010
H	2.606243	-4.063111	0.038108
H	2.468988	-5.854412	0.013536
H	4.052841	-2.233823	2.484922
H	0.462801	-4.461594	3.352960
H	-0.214746	2.306311	-5.358674
H	1.402861	3.076961	-5.168746
H	1.075857	1.970462	-6.558694
H	3.234652	1.664599	-4.424258
H	2.867807	-0.067744	-4.119391
H	2.943496	0.540552	-5.812300
H	-1.444139	-2.111545	5.290213
H	0.129289	-2.911935	5.650793
H	-0.447316	-1.539268	6.670342
H	2.153665	-1.697096	5.123705
H	1.971987	-0.085796	4.348483
H	1.610943	-0.271395	6.100287
H	0.082510	3.479061	4.302949
H	3.890219	2.161058	2.763007
H	4.361711	2.373487	-1.763427
H	0.942728	4.716197	-2.956586
H	-1.307355	5.516361	1.690066
H	0.125459	5.854055	2.726389
H	-0.106677	6.752396	1.179352
H	2.268377	5.095593	1.968428
H	2.322650	4.099306	0.474702
H	2.090851	5.874994	0.341456

204

Encapsulated P4(2-) butterfly

Si	3.575172	-4.849077	1.141750
Si	3.745872	-3.982766	-1.805299
Si	4.477895	0.336357	2.724850
Na	1.007375	-3.559092	0.042453
Na	3.662297	-1.621323	0.417820
N	-3.559268	-1.540249	-0.313004
N	3.243294	-3.805653	-0.175851
C	-4.875897	-1.368159	-0.225944

C	-5.939303	-2.424557	-0.665462
C	-6.643211	-1.848921	-1.911322
H	-7.129387	-0.885346	-1.708096
H	-7.410889	-2.554453	-2.266019
H	-5.922699	-1.696635	-2.728892
C	-6.974723	-2.649276	0.452591
H	-6.490237	-2.962788	1.389209
H	-7.671774	-3.446421	0.152390
H	-7.575633	-1.756629	0.670707
C	-5.404877	-3.804021	-1.063508
H	-4.699421	-3.756674	-1.898822
H	-6.257810	-4.418962	-1.389223
H	-4.916568	-4.331366	-0.238092
C	-2.859040	-2.759041	-0.458993
C	-2.660764	-3.580723	0.683375
C	-1.811185	-4.685998	0.570479
H	-1.647258	-5.328153	1.437794
C	-1.195006	-5.005998	-0.643368
H	-0.562242	-5.895132	-0.722270
C	-1.431381	-4.214145	-1.769037
H	-0.990306	-4.501692	-2.724793
C	-2.255390	-3.083098	-1.698454
C	-3.424516	-3.288686	1.964014
H	-4.415767	-2.931348	1.643999
C	-3.662915	-4.518951	2.846443
H	-2.692440	-4.937766	3.164261
H	-4.138072	-4.171175	3.778193
C	-4.531008	-5.605257	2.226911
H	-5.534413	-5.221609	1.985692
H	-4.658922	-6.453023	2.917003
H	-4.097103	-5.999220	1.295269
C	-2.814063	-2.145527	2.796027
H	-2.047568	-2.560514	3.472593
H	-2.258308	-1.459958	2.139840
C	-3.864816	-1.361236	3.566440
H	-3.408715	-0.598758	4.213293
H	-4.475339	-2.013759	4.210365
H	-4.545291	-0.850744	2.867689
C	-2.590721	-2.284237	-2.943419
H	-3.482669	-1.689917	-2.690171
C	-2.979505	-3.198753	-4.116158
H	-2.102682	-3.770832	-4.460525
H	-3.694863	-3.953076	-3.748527
C	-3.592366	-2.456456	-5.295036
H	-4.479800	-1.881208	-4.985319
H	-3.909344	-3.156990	-6.082344
H	-2.883307	-1.749903	-5.752483
C	-1.504025	-1.260346	-3.314273
H	-1.904183	-0.593333	-4.094261
H	-1.329032	-0.605208	-2.446420
C	-0.169328	-1.824637	-3.771540
H	0.532834	-1.012628	-4.011531
H	-0.265689	-2.454224	-4.669870
H	0.307172	-2.433739	-2.989356
C	2.131117	-4.707603	2.385251
H	1.864407	-3.661541	2.613196
H	2.387448	-5.190094	3.341822
H	1.223802	-5.224752	2.019986
C	3.668181	-6.689794	0.707454
H	2.769819	-7.004211	0.150770
H	3.736569	-7.309697	1.616624
H	4.544591	-6.917226	0.081831
C	5.167999	-4.398713	2.055879
H	6.031199	-4.534394	1.385058
H	5.325644	-5.027778	2.946992
H	5.175469	-3.346661	2.383918
C	2.432189	-4.904782	-2.823768
H	2.252262	-5.906360	-2.399215
H	2.730180	-5.031995	-3.877283
H	1.473494	-4.359309	-2.822785
C	5.386714	-4.888708	-2.055398
H	6.183837	-4.428087	-1.450503
H	5.700145	-4.849087	-3.111528
H	5.315564	-5.948735	-1.767536
C	3.945692	-2.270605	-2.596829
H	3.042391	-1.651369	-2.473894
H	4.146562	-2.347749	-3.677303
H	4.795322	-1.723171	-2.156368
C	3.158415	-0.961165	3.179250
H	3.556836	-1.987125	3.106887
H	2.249164	-0.903419	2.556125
H	2.830331	-0.830171	4.223223
C	5.922747	-0.007147	3.899003
H	6.431969	-0.949403	3.638771
H	5.563776	-0.093695	4.937908
H	6.670019	0.800221	3.869015
C	3.752592	2.013009	3.222385
H	4.490313	2.819966	3.095244
H	3.422870	2.018611	4.273983
H	2.877790	2.252922	2.598581
Si	3.060779	3.618742	-2.859769
Si	3.778158	4.874515	-0.108233
Si	6.383060	0.185393	0.302394
Mg	-2.177958	-0.013524	0.047334
Na	0.850359	3.889522	-0.670157
Na	3.639041	1.643869	-0.319002
N	-3.651280	1.386627	0.557560
N	3.071843	3.721392	-1.152606
N	4.845195	0.245696	1.054389
C	-4.948136	1.099964	0.593546
C	-5.457504	-0.159560	0.210790
H	-6.537045	-0.208393	0.263536

C	-6.050889	2.059195	1.141358
C	-6.563116	1.432146	2.454414
H	-6.978795	0.426966	2.302065
H	-7.349462	2.068403	2.890072
H	-5.749134	1.351855	3.190445
C	-7.216624	2.181949	0.142556
H	-6.867695	2.534213	-0.839549
H	-7.947318	2.913175	0.520492
H	-7.753445	1.236864	-0.013358
C	-5.597317	3.481758	1.480910
H	-4.801465	3.499261	2.231493
H	-6.457559	4.024643	1.901630
H	-5.249798	4.042132	0.607576
C	-3.040103	2.660409	0.610135
C	-3.037863	3.470756	-0.557051
C	-2.243114	4.621525	-0.564079
H	-2.231808	5.258692	-1.450740
C	-1.489921	4.991268	0.553287
H	-0.910307	5.919657	0.543611
C	-1.535675	4.209029	1.708174
H	-0.989054	4.535087	2.594413
C	-2.311736	3.044717	1.763128
C	-3.936936	3.117318	-1.731986
H	-4.853518	2.694037	-1.292523
C	-4.366643	4.326148	-2.571282
H	-3.474422	4.803424	-3.012238
H	-4.937913	3.947062	-3.433801
C	-5.211601	5.359481	-1.839168
H	-6.151130	4.916583	-1.473981
H	-5.477740	6.196484	-2.502478
H	-4.685998	5.780243	-0.968524
C	-3.358165	2.014860	-2.635550
H	-2.645843	2.466562	-3.347102
H	-2.749474	1.327881	-2.031614
C	-4.428242	1.211562	-3.358039
H	-3.982279	0.462063	-4.027401
H	-5.085548	1.846858	-3.972022
H	-5.059539	0.679637	-2.629634
C	-2.481308	2.274774	3.060210
H	-3.345819	1.611151	2.902137
C	-2.843583	3.212379	4.223773
H	-1.991496	3.869819	4.461223
H	-3.649216	3.887858	3.892011
C	-3.285614	2.480431	5.482976
H	-4.134547	1.808860	5.275327
H	-3.605586	3.189120	6.261783
H	-2.477154	1.868014	5.910275
C	-1.303336	1.343912	3.392395
H	-1.615111	0.661714	4.198993
H	-1.121181	0.690064	2.525397

C	0.001181	2.015477	3.787495
H	0.789626	1.265517	3.944424
H	-0.095380	2.593652	4.719731
H	0.365549	2.698984	3.006658
C	2.906115	1.812026	-3.406942
H	3.800655	1.218084	-3.160713
H	2.760819	1.736076	-4.496709
H	2.033305	1.341782	-2.924665
C	1.513146	4.486218	-3.565161
H	0.588756	3.955926	-3.270167
H	1.517986	4.510729	-4.667058
H	1.450209	5.530865	-3.213788
C	4.569433	4.349910	-3.730801
H	4.618498	5.442669	-3.608092
H	4.549353	4.131567	-4.811076
H	5.496256	3.921029	-3.316729
C	4.084878	6.585443	-0.858888
H	4.884864	6.553803	-1.614435
H	4.385541	7.309577	-0.083704
H	3.176542	6.972917	-1.349391
C	5.419760	4.278223	0.616258
H	5.337377	3.273726	1.060562
H	5.801665	4.955243	1.397383
H	6.178839	4.222855	-0.180919
C	2.569318	5.166357	1.342913
H	1.692154	5.762400	1.028963
H	3.042870	5.730121	2.162262
H	2.212258	4.217355	1.777223
C	6.230933	0.918292	-1.448244
H	5.975589	1.991813	-1.430339
H	5.471294	0.394178	-2.051340
H	7.182766	0.829588	-1.995559
C	7.780802	1.138711	1.146926
H	7.474309	2.169316	1.383837
H	8.661284	1.190879	0.485332
H	8.094571	0.652054	2.082392
C	7.000531	-1.597368	0.090994
H	7.195190	-2.046628	1.078079
H	7.935277	-1.644413	-0.491365
H	6.265495	-2.241988	-0.418366
P	0.945254	0.905545	0.778299
P	1.045389	-0.312565	-1.007659
P	0.010683	-1.120180	0.821322
P	-0.339241	1.437582	-0.983053

10.References

[1] B. Rösch, T. X. Gentner, J. Eyselein, J. Langer, H. Elsen, S. Harder, *Nature* **2021**, *592*, DOI 10.1038/s41586-021-03401-w.

[2] J. Maurer, L. Klerner, J. Mai, H. Stecher, S. Thum, M. Morasch, J. Langer, S. Harder, *Nat. Chem.* **2025**, *17*, 703–709.

[3] W. L. F. Armarego, *Purification of Laboratory Chemicals*, Butterworth-Heinemann, **2017**.

[4] S. Bachmann, R. Neufeld, M. Dzemski, D. Stalke, *Chem. – A Eur. J.* **2016**, *22*, 8462–8465.

[5] R. Neufeld, D. Stalke, *Chem. Sci.* **2015**, *6*, 3354–3364.

[6] A. Kreyenschmidt, S. Bachmann, T. Niklas, D. Stalke, *ChemistrySelect* **2017**, *2*, 6957–6960.

[7] Rigaku Oxford Diffraction, **n.d.**

[8] O. V. Dolomanov, L. J. Bourhis, R. J. Gildea, J. A. K. Howard, H. Puschmann, *J. Appl. Crystallogr.* **2009**, *42*, 339–341.

[9] G. M. Sheldrick, *Acta Crystallogr. Sect. A Found. Adv.* **2015**, *71*, 3–8.

[10] G. M. Sheldrick, *Acta Crystallogr. Sect. C Struct. Chem.* **2015**, *71*, 3–8.

[11] A. Thorn, B. Dittrich, G. M. Sheldrick, *Acta Crystallogr. Sect. A Found. Crystallogr.* **2012**, *68*, 448–451.

[12] M. J. Frisch, G. W. Trucks, H. B. Schlegel, G. E. Scuseria, M. A. Robb, J. R. Cheeseman, G. Scalmani, V. Barone, G. A. Petersson, H. Nakatsuji, X. Li, M. Caricato, A. V. Marenich, J. Bloino, B. G. Janesko, R. Gomperts, B. Mennucci, D. J. Hratch, **n.d.**

[13] A. D. Becke, *J. Chem. Phys.* **1993**, *98*, 5648–5652.

[14] J. P. Perdew, J. A. Chevary, S. H. Vosko, K. A. Jackson, M. R. Pederson, D. J. Singh, C. Fiolhais, *Phys. Rev. B* **1993**, *48*, 4978–4978.

[15] F. Weigend, *Phys. Chem. Chem. Phys.* **2006**, *8*, 1057.

[16] F. Weigend, R. Ahlrichs, *Phys. Chem. Chem. Phys.* **2005**, *7*, 3297.

[17] S. Grimme, S. Ehrlich, L. Goerigk, *J. Comput. Chem.* **2011**, *32*, 1456–1465.

[18] E. D. Glendening, C. R. Landis, F. Weinhold, *J. Comput. Chem.* **2013**, *34*, 1429–1437.

[19] R. F. W. Bader, *Chem. Rev.* **1991**, *91*, 893–928.

[20] T. A. Keith, *AIMAll Version 17.01.2025*, TK Gristmill Software, **2017**.
